# Shallow magmatic intrusion evolution below La Palma before and during the 2021 eruption

**DOI:** 10.1038/s41598-022-23998-w

**Published:** 2022-12-12

**Authors:** José Fernández, Joaquin Escayo, Antonio G. Camacho, Mimmo Palano, Juan F. Prieto, Zhongbo Hu, Sergey V. Samsonov, Kristy F. Tiampo, Eumenio Ancochea

**Affiliations:** 1 Instituto de Geociencias (CSIC, UCM). Calle del Doctor Severo Ochoa, nº 7, Ciudad Universitaria, 28040 Madrid, Spain; 2 Istituto Nazionale di Geofisica e Vulcanologia, Osservatorio Etneo - Sezione di Catania, Piazza Roma 2, 95125 Catania, Italy; 3 ETS de Ingenieros en Topografía, Geodesia y Cartografía, Universidad Politécnica de Madrid, 28031 Madrid, Spain; 4 Dares Technology, C/ Esteve Terrades, 1, Building RDIT Office 117, Parc UPC – PMT 08860, Castelldefels, Barcelona, Spain; 5 Canada Centre for Mapping and Earth Observation, Natural Resources Canada, 560 Rochester Street, Ottawa, ON K1A 0E4 Canada; 6 Cooperative Institute for Research in Environmental Sciences (CIRES), 216UCB, University of Colorado at Boulder, Boulder, CO 80309 USA; 7 Departamento de Mineralogía y Petrología, Fac. CC. Geológicas, Universidad Complutense de Madrid, 28040 Madrid, Spain; 8 Also at CommSensLab, Dep. Signal Theory and Communications, Universitat Politècnica de Catalunya (UPC), D3-Campus Nord-UPC, C. Jordi Girona 1-3, 08034 Barcelona, Spain; 9 Present Address: Instituto de Geociencias (CSIC, UCM). Calle del Doctor Severo Ochoa, nº 7, Ciudad Universitaria, 28040 Madrid, Spain

**Keywords:** Natural hazards, Solid Earth sciences

## Abstract

La Palma, Canary Islands, underwent volcanic unrest which culminated in its largest historical eruption. We study this unrest along 2021 using Interferometric Synthetic Aperture Radar (InSAR) and a new improved interpretation methodology, comparing achieved results with the crustal structure. We reproduce the final phase of La Palma volcanic unrest, highligthing a shallow magma accumulation which begins about 3.5 months before the eruption in a crustal volume charactherized by low density and fractured rocks. Our modeling, together with our improved pictures of the crustal structure, allows us to explain the location and characteristics of the eruption and to detect failed eruption paths. These can be used to explain post-eruptive phenomena and hazards to the local population, such as detected gases anomalies in La Bombilla and Puerto Naos. Our results have implications for understanding volcanic activity in the Canaries and volcano monitoring elsewhere, helping to support decision-making and providing significant insights into urban and infrastructure planning in volcanic areas.

## Introduction

**Figure 1:**
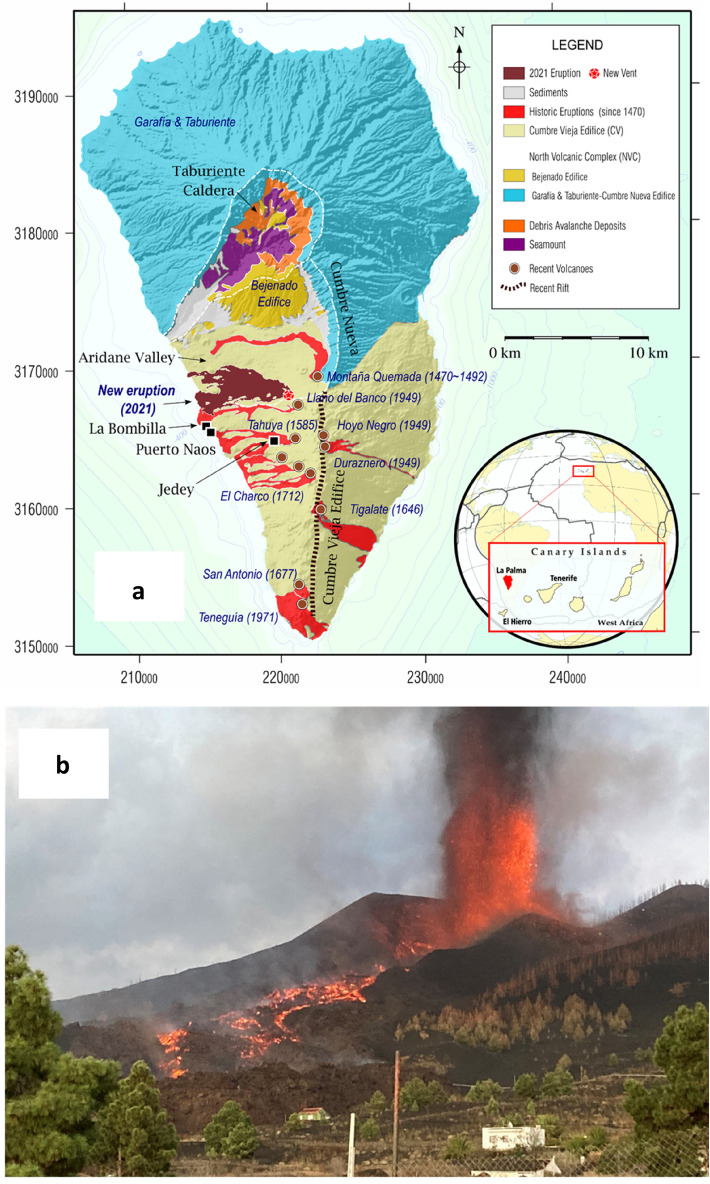
Geographic location and geologic elements of La Palma and eruption photo. (**a**) Location of Canary Islands and La Palma Island (lower inset) and simplified updated geological map (UTM28 North reference system, coordinates in m). The main volcanic complexes, ridges as well as the last historical eruptions on the Island are shown. The towns of Jedey, La Bombilla and Puerto Naos (black squares) are also depicted. The 2021 lava flow field was the most voluminous of the last 600 years. See upper insets for scale and keys description. (**b**) Snapshot of the eruption during the first days after September 19, 2021. Photo by the authors (September 23, 2021; 20:12 local time). GMT software (www.generic-maping-tools .org) and Microsoft PowerPoint 2016 were used to create this figure.

La Palma island (Fig. 1) has one of the highest potential volcanic risks in the Canaries as demonstrated by its historic unrest^1–6^, and the subsequent eruption^7,8^ that began on September 19th, 2021, on the western slope of Cumbre Vieja ridge. In the following days, intense effusive and explosive activity^8–10^ occurred. This activity devastated human settlements with a cost > 900 M€ and forced the evacuation of more than 7000 residents^11^. Eruptive vents opened, collapsed and closed, grouping around a ~ 740 m long fissure trending NW-SE. The eruption ended^9^ on December 13th, 2021. Although seismic activity in La Palma was not observed prior to 2017 (Supplementary Fig. 1), nine seismic swarms^12^ (Supplementary Table 1) were registered between 2017–2021. The last, on September 11–19, 2021, heralded the onset of the largest historical eruption in La Palma.

La Palma (Fig. 1) and El Hierro are the westernmost islands of the Canaries. La Palma rises from the ocean floor about 4000 m deep and reaching a height of ca. 2500 m above sea level. It has been volcanically active for at least four million years and has had 7 eruptions in the last 500 years, including the 2021 one.

The island has an approximately triangular shape and consists of two large volcanic bodies: the older one located to the North consist of a large, more or less circular, volcanic complex (Northern Volcanic Complex, NVC), with a central depression (Taburiente Caldera), and a younger elongated volcano, developed along the southern portion of the island^3^ (Fig. 1).

NVC is formed by several superimposed volcanic structures (Garafía, Taburiente, Cumbre Nueva and Bejenado edifices, 1.7–0.4 Ma). The southern region has been destroyed by large landslides and intense erosive processes. Those processes gave rise to the Taburiente caldera^13,14^, at the bottom of which we can observe rocks from the island’s submarine growth phase (3–4 Ma), currently elevated to 1500 m of altitude, as well as plutonic rocks formed by the multiple magmas that rose and cooled in the crust.

The southern part of the island is formed by the younger edifice (Cumbre Vieja, CV), at almost 2000 m of altitude, elongated ca. 17 km along the approximate NS direction, and extending several more kilometres under the sea. All recent on-land volcanic activity has taken place in this edifice, also including all historical eruptions (Fig. 1). See Fernández et al.^3^, and references therein, for a more complete geological and volcanological description.

Considering the previously existing unrest^1–6^ which finished with the 2021 eruption, we use InSAR time-series to look at changes in deformation over time from January 2021 until the eruption, and use a new improved methodology^3,15^ to perform time-series inversions to determine the best-fitting sources that explain the deformation. In this methodology, the observed ground deformation patterns are modeled by 3D deformation sources (as a combination of pressure and dip-slip, strike-slip and tensile dislocation sources) located below surface.

The inversion methodology used corresponds with that described by Camacho et al.^15^ and improved by Fernández et al.^3^ incorporating the formulation of Geertsma and Van Opstal^17^ and of Okada^18^ for pressure and dislocation sources respectively. We have improved here this methodology (Methods section) by introducing the use of line-of-sight (LOS) displacement data from InSAR and sequential monitoring data, together with the assumption of possible existence of offset values in the data sets. In our calculations we use decimal fractions of years, but for the description of results we also use the typical date format facilitating reading and comparison of results.

The deformation that occurred before and during the onset of the 2021 eruption was studied by de Luca et al.^19^, but our study extends the analysis period and the modeling approach allow for both a spatial reconstruction and more detailed temporal sequence of the observed deformation sources.

Finally, we compare the fitted sources to updated crustal structure results to gauge the effect of this on preferred magma pathways beneath La Palma. The crustal structure of La Palma has been studied previously^3,20–22^, however in this study we provided a significant update. The main feature of the previous models^3,20–22^ is the existence and morphology of a very large and dense intrusive body located below the NVC corresponding to dike swarms and accumulated plutonic material. This outstanding structure is responsible for a gravimetric anomaly variation of about 130 mGal. Considering that this large anomaly associated with the NVC of the Island can mask or distort other anomalies of both smaller magnitude and/or extent on the island, we tested the option of using only the data from the southern part of the island, applying the same gravimetric inversion methodology^3^ but to the data reflected in Supplementary Fig. 4.

Our objective is to obtain a clear description of the spatio-temporal evolution of the shallow intrusion process and the onset of the eruption, which in turn would allow us to provide a set of parameters to timely constraint the location, geometry, orientation and size of potential shallow magmatic storages. Combining the inversion results with the cortical structure model, we obtain information on the possible location of the eruption, assisting with the discrimination between fissure and central eruptions. All these aspects will be useful in future events in La Palma, the Canary Islands and elsewhere.

## Results

**Figure 2:**
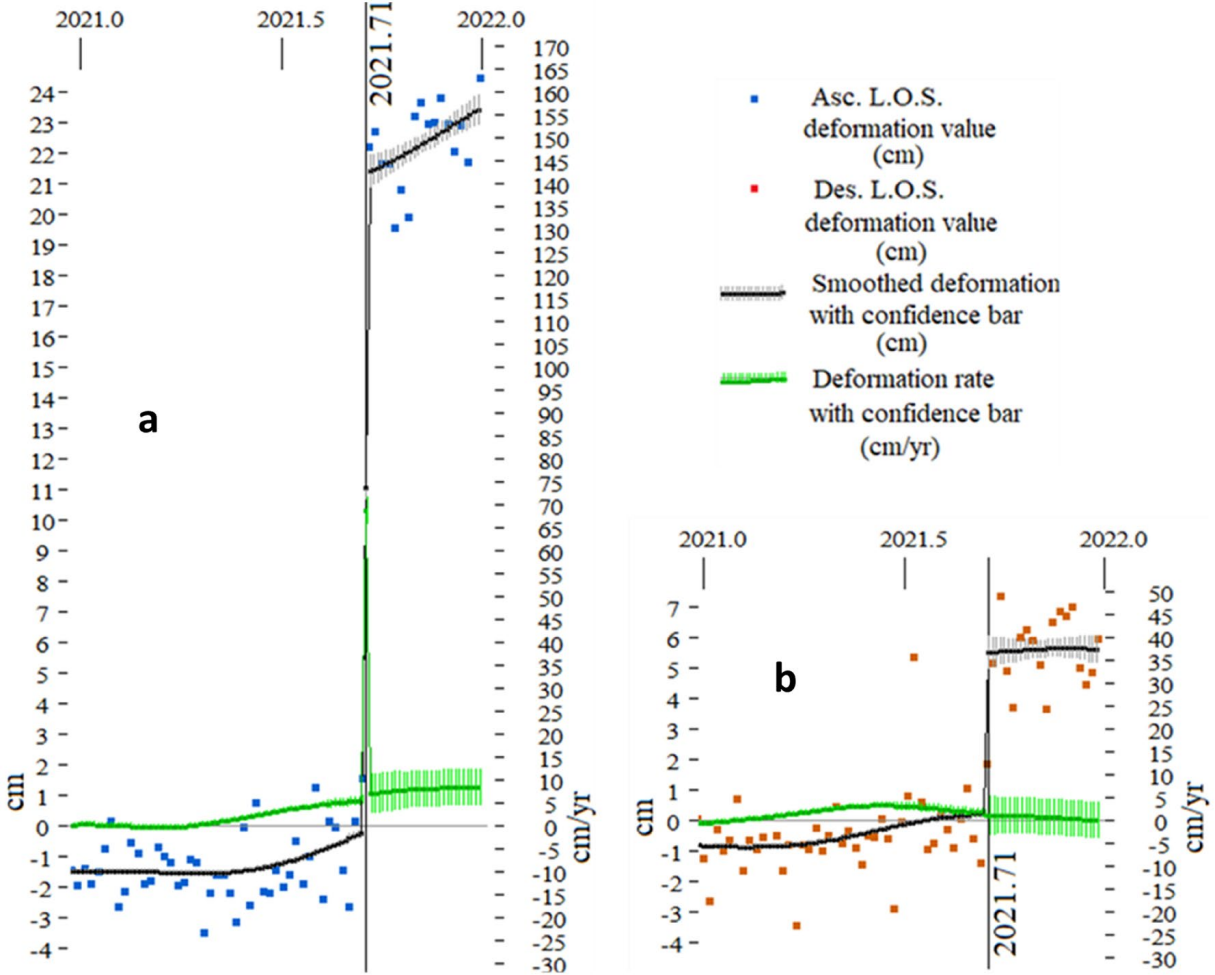
Input deformation data for a pixel close to the maximum deformation site with UTM coordinates (218,000,3,166,000). Dots denote the discrete satellite data, using blue color for ascending (**a**) and red for descending (**b**) LOS displacements. Black lines are smoothed deformation (cm) (left axis) with confidence bars for one standard deviation. Smoothing approach is described in the Methods section. Green lines are smoothed deformation rate (cm/yr) (right axis) with confidence bars for one standard deviation. At 2021.71 (09/17/2021) there is a clear jump in the deformation record. Pixel location is shown in Supplementary Fig. 3. Matlab software (www.mathworks.com) and Microsoft PowerPoint 2016 were used to create this figure.

For the InSAR analysis, we produced InSAR time series over La Palma from 2017–2021 using all available data from the ESA’s Sentinel-1A/B constellation, with atmospheric phase screen corrections (see details in Methods). We obtained mean LOS velocities (Supplementary Fig. 2) and displacement time series (Fig. 2 and Supplementary Fig. 3) for each ascending and descending satellite orbit. Supplementary Fig. 3 shows LOS displacement time series for selected pixels, compared with the LOS projected GNSS displacements for the stations available on La Palma island^12,19^. Good agreement is observed between both techniques, validating the InSAR results.

One important characteristic of the observed deformation (Fig. 2 and Supplementary Fig. 4) is that it increases very sharply, shortly before the onset of the eruption. Therefore, considering our main objective and that the unrest onset was studied previously^3^, we concentrate our study in 2021, the final unrest period before the eruption, searching for possible precursory signals in the deformation modeling results, which could be useful to support decision-making in future events.

**Figure 3:**
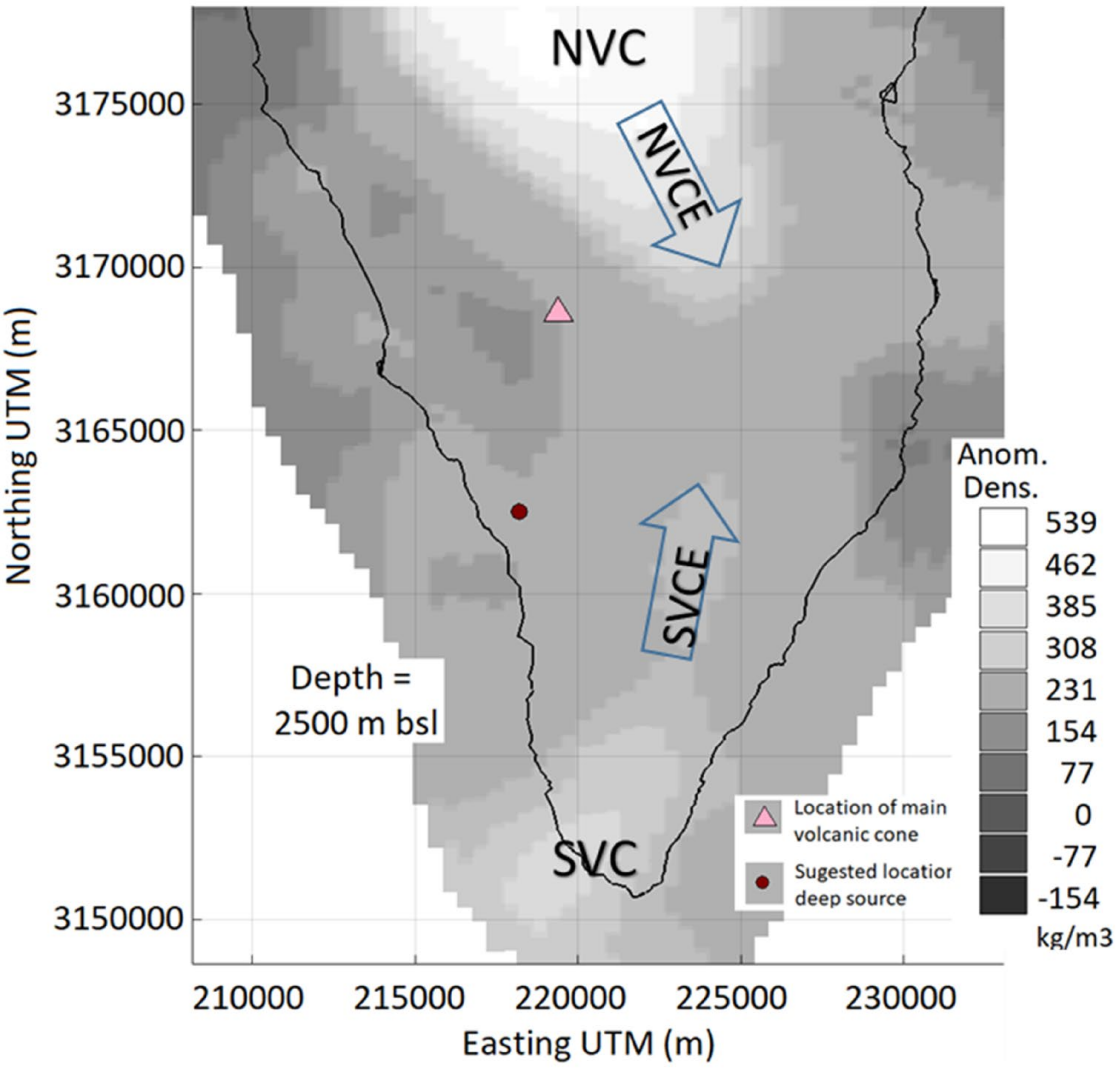
Horizontal slice of the obtained 3D crustal density model for La Palma. Horizontal section of the 3D model for anomalous density for La Palma Island at 2500 m depth below sea level (bsl). Suggested location of deep source is an approximation to the location of the input of magma from deeper zones, from our geodetic modeling. Matlab software (www.mathworks.com) and Microsoft PowerPoint 2016 were used to create this figure.

**Figure 4:**
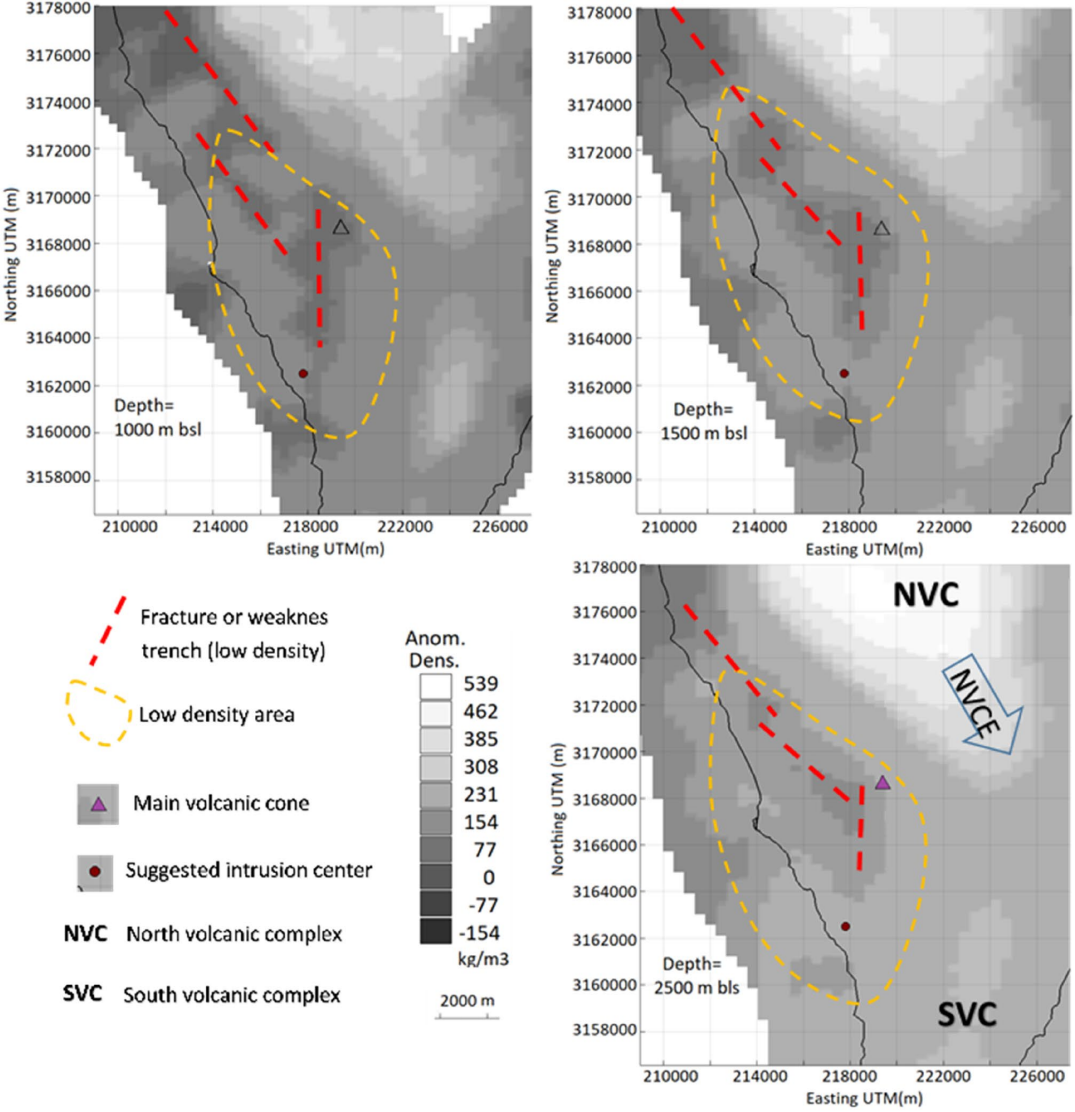
Density crustal model for La Palma Island. Horizontal sections of the crustal density model for La Palma Island at different depths bsl. Density minima alignments, in particular associated with the shallow magma storage and the fissure eruption, are marked with red dashed lines. Triangle indicates the location of the main volcanic vent during the eruption. Red point indicates the location of the deep magma feeding the shallow magmatic reservoir below Jedey area from our geodetic modeling. Low density area is approximately delineated by the dashed yellow line. Matlab software (www.mathworks.com) and Microsoft PowerPoint 2016 were used to create this figure.

An important information to frame and interpret deformation measured in volcanic areas is the crustal structure^23,24^. Valuable crustal structure information comes from the gravity data modeled by using a fitted 3D model for anomalous subsurface densities. We employ this at La Palma Island using a structural gravity inversion methodology^25^ which considers stratified structures with density increases with depth along with the available gravity data (Supplementary Fig. 5). In the modeled crustal structure we identify some interesting features related to the more recent volcanic activity (most of the South part of the island is younger than 20–36 Ky^26,27^). Figure 3 shows a horizontal section of the adjusted 3D model for the studied sector of the island. Figure 4 shows some details from the region of the intrusion and the eruption in the CV area.

LOS deformation time series for 2021 were modeled to constrain the location and geometry of the magmatic plumbing system (MPS) and its temporal evolution using the new inversion methodology^3,7^ discussed in Methods. 3-D arbitrary sources for pressure and dislocations (strike-slip, dip-slip, and tensile) are adjusted. No particular hypotheses about the nature of the source (pressure, dislocations), shape or location are required. The inversion methodology provides results for deformation sources as 3D cell aggregations to which the inversion process automatically assigns a source type, magnitude values (MPa for pressure and cm for dislocations), position, and orientation (angles of dislocation planes). The non-linear problem is solved by the explorative approach, and the ambiguity of the problem is solved by the addition of simple regularization conditions about the total magnitude of the anomalous structures (the addition of all strength values for the adjusted sources). Planar, exact or homogeneously distributed data are not required.

We focus our study in the CV area around the eruption zone, searching for spatial–temporal details of the deformation sources and the MPS. Ascending and descending LOS displacement data are inverted simultaneously. Considering the small number of GNSS stations^9,28^ (7 in the island, and just 2 close to the main deformation area, see Supplementary Fig. 3) and that, as a consequence of this, the effect of these data in the inversion results will be null^29^, only InSAR data are used for inversions.

The visual inspection of the LOS time series defines 3 different periods, charactherized by different deformation patterns (Fig. 2). The first period spans from 2021.00 to 2021.70 (01/01/2021–09/13/2021), representing the pre-eruptive stage, and is charactherized by a moderate magnitude of the LOS deformation (few cm) coupled with moderately low noise. The second period captures the shallow intrusion onset (2021.70–2021.72, 09/13/2021–09/20/2021) and shows a significant jump, both in ascending (~ 21 cm in the case plotted in Fig. 2A) and descending (~ 7.5 cm in Fig. 2B) LOS data. The last period covers the 2021.72–2022.00 (09/20/2021–01/01/2022) time interval, sampling the post-intrusion deformation, which is characterized by a complex pattern (see Fig. 2). Given these results, we have carried out the time series deformation data inversion for three time intervals: (1) 01/01/2021–09/13/2021; (2) 09/13/2021–09/20/2021; and (3) 09/20/2021–01/01/2022.

**Figure 5:**
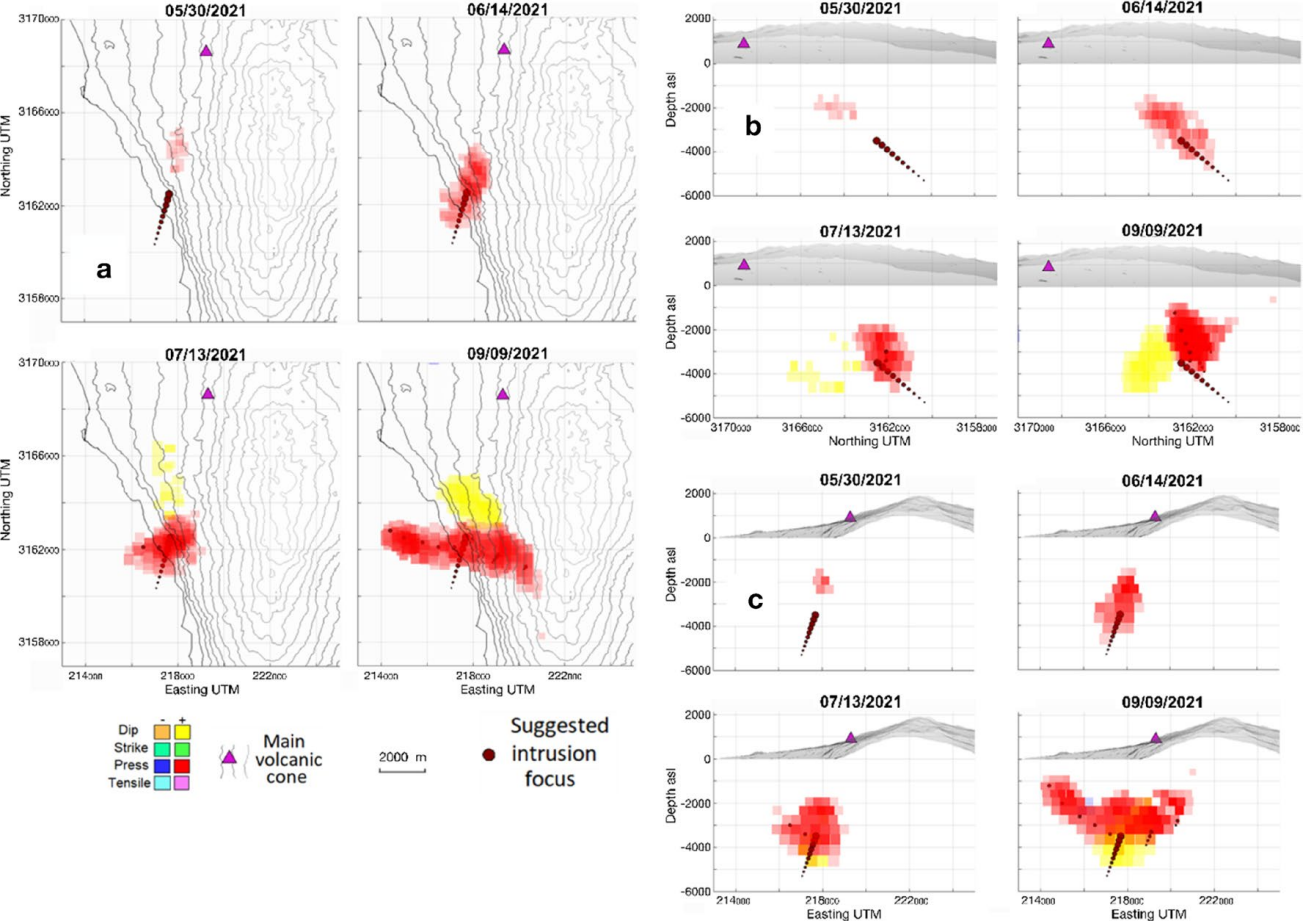
Time evolution of the modeled sources for 01/01/2021–09/13/2021 (2021.00–2021.70). (**a**) Horizontal, (**b**) vertical N-S and (**c**) vertical W-E projections of the sources obtained for different epochs in 2021.00–2021.70. The different plots show the time evolution of the different sources obtained from inversion of Sentinel-1 ascending and descending LOS displacement. Purple triangle shows the location of the main volcanic vent during the 2021 La Palma eruption. Red dots indicate the provenance of the deep magma feed in the detected shallow reservoir from the geodetic modeling. All coordinates and depths are in meters. Matlab (www.mathworks.com) and GMT (www.generic-mapin-tools.org) softwares were used to create this figure. This figure complements with Supplementary Movies 1–6.

For all the intervals we use the same selection of pixels (3992 pixels, mutually separated by distances ≥ 290 m) with ascending and descending data. And for all of them we use the same 3D partition of the cells, with an average side of 350 m. In general, the size of the cells does not affect the general characteristic of the adjusted model. However, to avoid any possible size-dependent artifacts we always use cells with sizes larger than the horizontal distance between neigbouring data pixels. We performed a set of checkerboard tests to ensure that the resolution of the InSAR results is sufficient for the inversion (see Supplementary Information). One example is shown in Supplementary Fig. 6. Modeled sources and characteristics are shown in Figs. 5–8, Supplementary Fig. 7 and Supplementary Movies 1–6.

## Discussion

Compared to previous studies^3,20,21^ of the on-land 3D density structure, we provide more information on the existence and morphology of anomalous structures in the southern half of the island. In particular, we identify the existence of a clear extension of NVC towards the southeast (NVC extension, NVCE), partially below CV (Fig. 3). We also observe the presence of an incipient Southern Volcanic Complex, SVC, located outside the island to the southwest, as well as its possible northeast extension, SVCE, again approaching CV. In addition, we observe an alignment of density minima on the western slope which went unnoticed in previous studies, overshadowed by the large anomaly of the NVC (Figs. 3–4). A more detailed discussion on the structural results is given in the Supplementary Information.

We summarize the inversion results of LOS displacements and their time evolution (see Figs. 5–9 and Supplementary Movies 1–6), considering the three time periods discussed above:

1. **Eruption run-up, 01/01/2021–09/13/2021:** The most relevant feature of this period is the appearance and growth of a magmatic intrusion, shown mainly by a source of positive pressure of increasing intensity (Fig. 5), located about 5 km SSW of the main eruptive vent of the incoming eruption, in the area near the town of Jedey (Fig. 1). It clearly shows the filling of a shallow magmatic reservoir. This phenomenon begins around 2021.40 (05/26/2021), about 3.5 months before the eruption and, considering the small intensity/volume of the pressure source, probably is caused by the contact of an ascending dike with the existing hydrothermal system in the area associated with CV^30,31^. The previous seismic activity^3,9^ (e.g., several seismic swarms in the second half of 2020 and in 2021.10 [02/06/2021]; Supplementary Table 1), were probably associated with the opening of the path for magma ascent from deeper zones. They were mostly located in the depth range 10-25 km^30^, and are significant in that they are partially located below the detected pressure source, see Supplementary Fig. 8. This source starts manifesting itself at a depth of 2.5 km, but over time, as it intensifies, it increases in extension and depth, elongating towards the ocean. In the first phases of the intrusion, the ascent of magma would take place in small amounts through pre-existing cracks or cracks formed by the registered seismic activity, taking advantage of volumes in the most superficial crust of the island composed of porous and unconsolidated material. This could be the reason that there is not much shallow seismicity (depth < 10 km) before the last seismic swarm and the eruption onset (Supplementary Fig. 9). More than a month before the eruption (~2021.55, 07/20/2021), incipient positive dip-slip sources activated (shown as yellow sources in Fig. 5) at a depth of about 3 km, suggesting a brittle response of the shallow crust to the rising of magma. As of 2021.55, an increase in intensity is detected in the deepest zone of the pressure source, as a consequence of the large inflow of magma (Fig. 5). The positive dip-slip sources also are associated with an elevation of the limiting block with the fault/main fracture zone. The modelled increase in pressure/volume in the shallow hydrothermal system is related to the fast influx of deeper magma. In addition, it seems that this intrusion occurred about 7 km SSW of the main eruptive fissure, in the area near Jedey (Fig. 1). The location of the shallow reservoir in a low-density zone, with fractured and/or porous material, facilitate this magma storage accumulation^30^. The total strength increases from 50 MPa km^3^ at 2021.45 (06/14/2021) up to 150 MPa km^3^ just before the eruption. The line followed by the rising magma corresponds to a pre-existing fracture or line of weakness (see Fig. 4), which structurally controlled the onset of the eruptive fissure at the surface (Supplementary Fig. 7). Our results for the pre-eruptive period (1) are consistent with those obtained by De Luca et al.^19^, although our results are more detailed and complete because we apply time series inversion and a new modeling tool which adjusts the free 3D geometry of the sources^3,15^.
2. **Eruption onset, 09/13/2021–09/20/2021:** In this period, contemporaneously with the development of the seismic swarm (Supplementary Table 1) preceding and accompanying the eruption onset, a large magma intrusion occurs (Fig. 6) This magmatic intrusion occupies a volume much larger than that of the previous period and is characterized by at least three ascending branches that probably take advantage of preexisting structural weakness lineaments in the crust (Fig. 4). In the second half of this period, magma reached the surface arising along the northernmost branch and led to the formation of the eruptive fissure (Fig. 6). This event occurred contemporaneously with a strong fracturing process, as suggested by the strike-slip sources located in a wide depth range (Fig. 6), going from the surface to 6 km, probably associated with the seismic swarm. The most superficial strike-slip sources (≤ 0.5 km bsl) highlight the path followed by the magma during its rise to the eruptive fissure at the surface.

**Figure 6:**
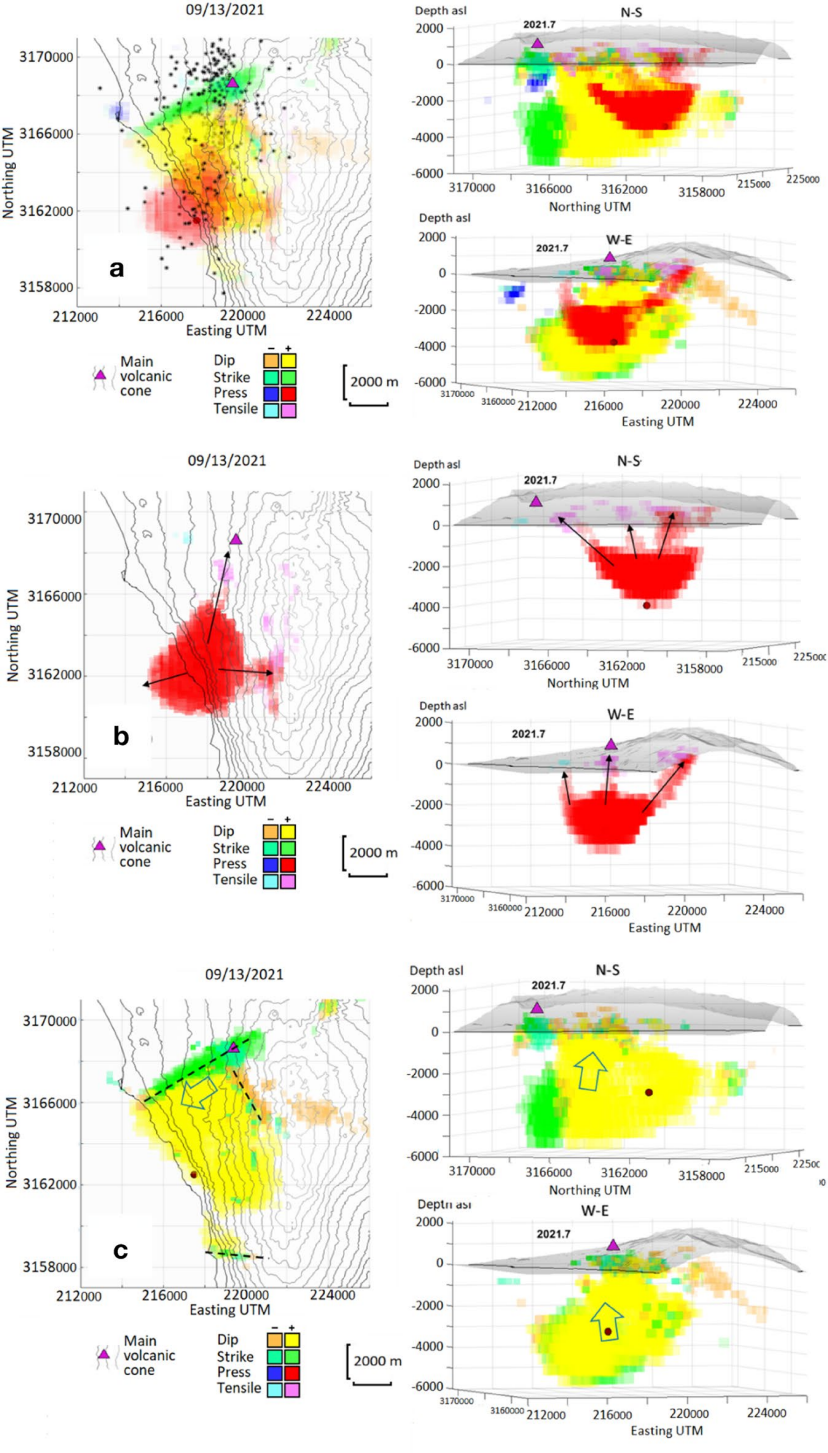
Time evolution of the modeled sources for 09/13/2021–09/20/2021 (2021.70–2021.72) period. (**a**) Horizontal and vertical N-S and W-E projections of the sources obtained for the jump in displacements measured in 2021.70–2021.72. Black stars represent the seismicity in that period. (**b**) Horizontal and vertical N-S and W-E projections of the pressure and tensile sources obtained for the jump in displacements measured in 2021.70–2021.72. (**c**) Horizontal and vertical N-S and W-E projections of the dip- and strike-slip sources obtained for the jump in displacements measured in 2021.70–2021.72. The different sources are obtained from inversion of Sentinel-1A/B ascending and descending LOS displacement. Purple triangle shows the location of the main volcanic vent during the 2021 La Palma eruption. Red dot indicates the provenance of the deep magma feed in the detected shallow reservoir. All coordinates and depths are in meters. Matlab (www.mathworks.com) and GMT (www.generic-mapin-tools.org) softwares were used to create this figure. This figure is complemented by Supplementary Movies 1–6.
  In the pressure structure, in addition to the main branch associated with the eruptive fissure, two other ascending branches are activated. These two branches are located underwater, in the area south of Puerto Naos, and to the East of Jedey (see Figs. 1 and 6). They do not reach the surface and therefore can be considered as failed magmatic intrusions. In Fig. 6b, where only the pressure sources are shown, we indicate with black arrows the three active ascending paths. Conceptually, these branches should be dikes and, if we observe in detail the sources which compose them, their mechanism is tensile in the upper portion, as would be expected for subsurface dikes. These details are best appreciated in panel b of Fig. 6 and Supplementary Fig. 7. In Fig. 6c, where only dip- and strike-slip dislocations are shown, the displacement directions are indicated with arrows, and the rupture lines with broken lines. The dip-slip sources cover a range of depths similar to that of the strike-slip sources, occupying a large volume around the magmatic intrusion, reflecting the process of magma rising from deeper regions and the consequent elevation of that structural block caused by the intense ascending magma flow just before the start of the eruption. This configuration of the shallow MPS combined with the fractures of the shallow crust can probably explain the persistence of the gas emission existing in the inhabited areas of Puerto Naos and La Bombilla (Fig. 1), six months after the end of the eruption^32^, which have prevented the return of its inhabitants. Volcanic unrest in the CV area has been associated with landslide and tsunami hazards^33^. Our results for InSAR and modeling (Supplementary Fig. 2 and Fig. 6c) clearly demonstrates slope movement characterized by both, dip-slip and strike-slip movements, showing a clear evidence that the intrusion associated with this eruption destabilized the western flank of CV, but did not lead to collapse. Therefore, larger or sucessive intrusions, or a different mechanism, is likely necessary to produce collapse, suggesting the need for additional research^34^. Again, our results for the co-eruptive period (2) are consistent with that obtained by De Luca et al.^19^ and the differences are the result of the different modeling tool employed here^3,15^.
3. **Co-eruptive deformation, 09/20/2021–01/01/2022:** The modeling of the time series displacements in the later stage reflects the co-eruptive sources (until 2021.95, 12/13/2021) and the start of the post-eruptive stage (2022.00, 01/01/2022), Figs. 7–8. Most of the sources in these stages are very shallow (depth ≤ 2 km). We have tensile sources in the co-eruptive phase but there is not new massive magma accumulation in the shallow reservoir, supporting the hypothesis that, after the first days of the eruption, magma comes directly from deeper zones^35^. However, a small shallow storage formed between Jedey and Puerto Naos, at about 2 km depth (Fig. 7) which stopped its activity at the end of November (~ 2021.90, 11/25/2021). Dip-slip sources again should be related with magma ascent, decrease temporally, being replaced by strike-slip sources that may be more related to fractures associated with feed system adjustment processes at the end of the eruptive and beginning of the post-eruptive stages^30^. The eruption finish in 2021.95 (12/13/2021) (Fig. 8) and although small tensile sources appear subsequently, they do not reach the surface (Fig. 8).

**Figure 7:**
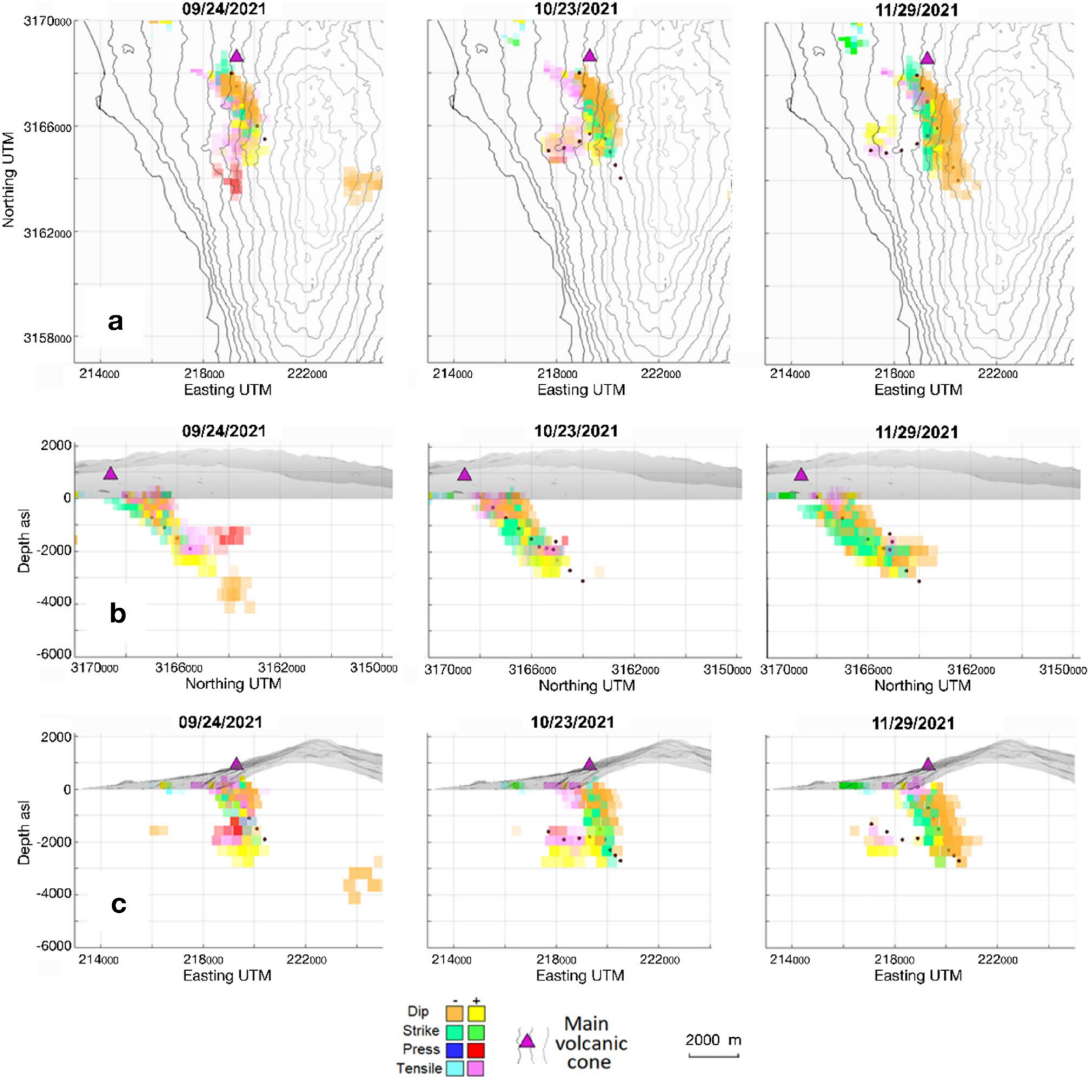
Time evolution of the modeled sources for 09/24/2021–11/29/2021 (2021.73–2021.91). (**a**) Horizontal, (**b**) vertical N-S and (**c**) vertical W-E projections of the sources obtained for diferent epochs in 2021.73–2021.91. The different plots show the time evolution of the different sources obtained from inversion of Sentinel-1 ascending and descending LOS displacement. Purple triangle shows the location of the main volcanic vent during the 2021 La Palma eruption. Red dots indicate the inferred direction of magma flows from deeper areas. The isolated negative dip-slip source which only appear in 09/24/2021 to the East of the vent could be real or an artifact produced by the location to the limit of the study area. But with the information available we can not discriminate between both options. Matlab (www.mathworks.com) and GMT (www.generic-mapin-tools.org) softwares were used to create this figure. This figure complements with Supplementary Movies 1–6.

**Figure 8:**
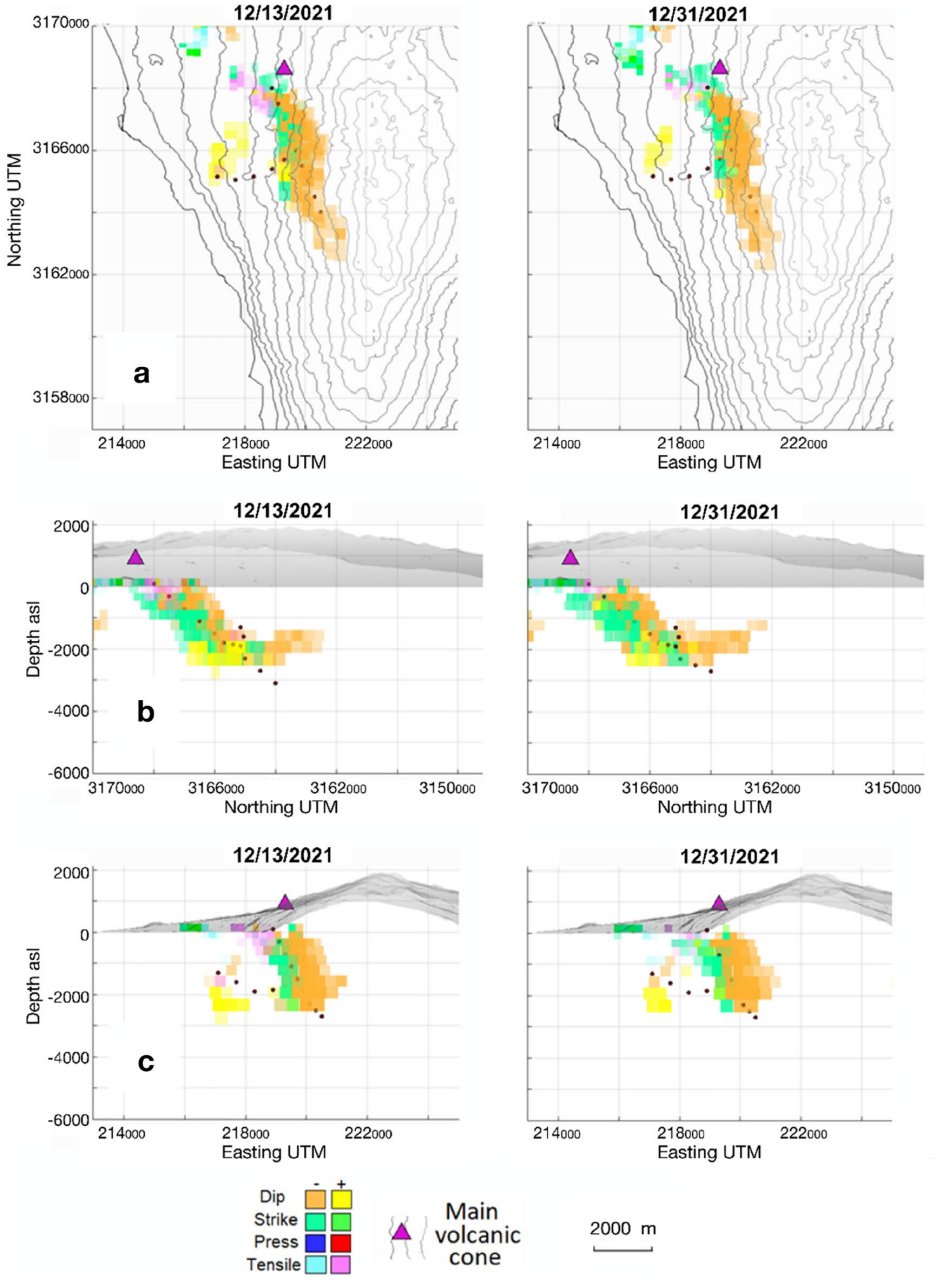
Time evolution of the modeled sources around the end of the eruption. (**a**) Horizontal, (**b**) vertical N-S and (**c**) vertical W-E projections of the sources obtained for different epochs in 2021.95 (12/13/2021, end of the eruption) and 2021.99 (12/28/2021, one of the initial post-eruptive steps). The different plots show the time evolution of the different sources obtained from inversion of Sentinel-1 ascending and descending LOS displacement. Purple triangle shows the location of the main volcanic vent during the 2021 La Palma eruption. Red dots indicate the inferred direction of magma flows from deeper areas. Matlab (www.mathworks.com) and GMT (www.generic-mapin-tools.org) softwares were used to create this figure. This figure complements with Supplementary Movies 1–6.

Previous studies indicate that short-term storage reservoirs formed at shallow depths (2–5 km) weeks-to-days before the eruptions^36,37^. Our results show that the shallow accumulation of magma below Jedey (at ~ 1–5 km) started ~ 3.5 months before the eruption, which can be related with the earliest eruption products^35^. This shallow reservoir is consistent with previous results^38^ highlighting the primary role played by CV structure in favouring the accumulation of magma in shallow reservoirs before the eruption, as well as with the shallow structure obtained from seismic tomography^30^. But the seismic tomography results^30^ do not detect this shallow magmatic reservoir and they postulate that magma ascended from 10 km depth to the surface in less than 10 days. This short-term shallow storage before the eruption has implications for volcano monitoring on the island.

Comparing with the crustal structure (Fig. 4), we observe two relevant features of interest: (1) a general zone of low relative density, marked with a yellow dashed line and bounded by the two volcanic complexes (NVC and SVC), and (2) very low relative density alignments (red dashed lines). The first would be a volume outside the existing high-density intrusive complexes made up of volcanic and sediments deposited on the oceanic crust. The red alignments reveal possible trenches or fracture lines parallel to the edges of the intrusive complexes. These could represent a low-density zone that is likely to house aquifers^31^ and to serve as a channel for the propagation of intrusions. We observe that shallow accumulation of magma (pressure sources) occurs in a fractured and unconsolidated volume of low density, and likely high porosity, capable of housing and transmitting fluids. This area coincides with the zone of structural weakness delineated by seismic tomography^30^, a highly fractured and brittle hydrothermal zone, which theoretically offers low resistance to magma ascent. This location of the shallow magma reservoir highlights the importance of understanding the structural model of the island in conjunction with the paths followed by magma in recent eruptions. We also detected that the final eruptive fissure opened along an alignment of density minima (Figs. 4 and 6 and Supplementary Fig. 7). Both results have important implications for volcano monitoring, but also are important for future urban and infrastructure planning in La Palma, and in volcanic archipelagos more generally, for characterization of hazard and risk mapping, and for the successful, sustainable and resilient urban reconstruction of La Palma island after the end of the eruption.

Our results allow us to propose a conceptual model for the pre- and co-eruptive shallow magma storage. First, considering results from Fernandez et al.^3^ and the results described above, we suggest that magma coming from depth^2,39^ attempted to follow several paths to the surface, that appear to be associated with the more recent eruptions, as the central boundary zone between NVC and CV^3^ and the Jedey zones of the island (Fig. 1). This result confirms previous suggestions^40^ that pre-existing intrusion zones represent a preferred pathway for uprising magma because of their weaker and stressed hosting rocks. Magma likely moved in the 8–14 km depth range, the zone correspondings to the lower crust beneath La Palma, the pre-island middle seafloor, representing a regional horizon of neutral buoyancy^36,38,41,42^ for Canarian magmas. There magma stalls in crustal reservoirs for years/decades before a possible eruption^42^ (in this case, more than a decade since the volcanic unrest onset of 2009–2010^3^).

This zone, along the sloping boundary separating the NVC and CV edifices^3^ and SVC, is where magma moved, altering the hydrothermal system and/or the aquifers, until it focused in the weak zone for shallow storage below Jedey (Fig. 5). Additionally, a second crustal magma reservoir could be located at this depth range (8–14 km), in agreement with previous volcanic activity^42^, with precursory and co-eruptive seismicity^7^, and the seismic tomography results^30^.

**Figure 9:**
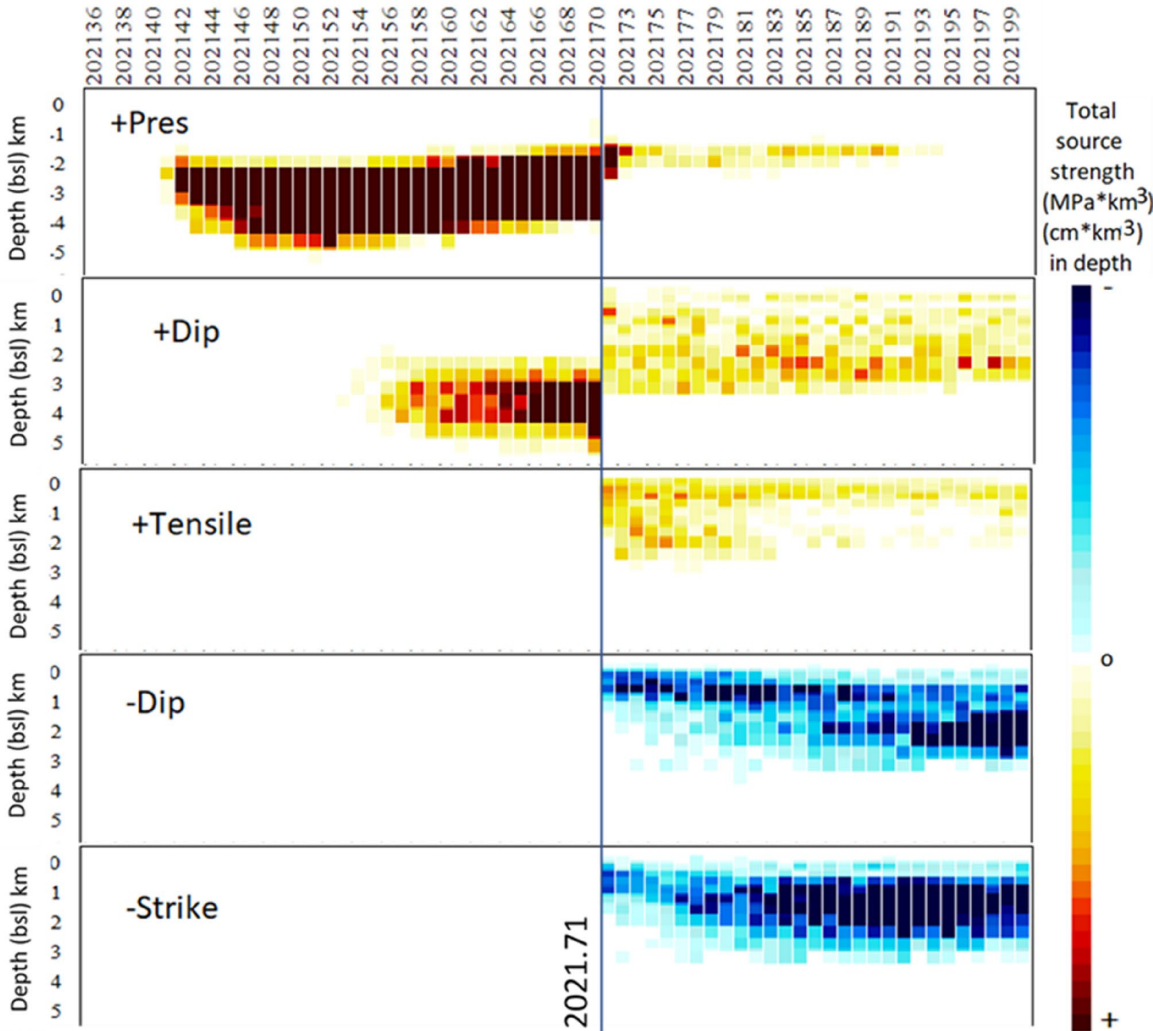
Graphical evolution of the characteristics of the sources below Jedey area. Panels show intensity chronograms as function of time (x axis) and depth range (km, y axis). The evolution of the different types of considered sources before and after the “jump” episode (2021.71, 09/17/2021) in deformation are indicated using hot colours (from yellow to garnet) to show increasing positive values, and cold colours (from light to dark blue) for increasing negative values. Pressure strength values range between ± 20 MPa·km^3^ each km in depth, dislocation strength values range between ± 50 cm·km^3^ each km in depth. Two precursor phenomena are clearly distinguished: (1) the intrusive pressure increase from 2021.40 (05/26/2021), and (2) the ascending mass dip from 2021.55 (20/07/2021). Matlab software (www.mathworks.com) was used to create this figure.

If we compare the chronograms (intensity-depth-date) of the various types of deformation sources (pressure and tensile, strike- and dip-slip), Fig. 9, we observe that the first thing that occurs in the study area, and after the fracturing produced by the seismic activity^3^ of 2020 and begining 2021 (Supplementary Table 1), is the intrusion (in 2021.40, 05/26/2021). Results show a pressurizing source, which represents the accumulation of magma in a shallow reservoir with an average depth of 3.5 km and located in one of the zones of recent volcanic activity and weak crustal structure (Figs. 1 and 5). In 2021.55 (07/20/2021) positive dislocation sources appear, probably associated with the beginning of a larger magma inflow, coinciding with the first days of the seismic swarm that would accompany the beginning of the eruption. The shallow dip- and strike-slip fractures related to the formation of the eruptive fissure appear in 2021.70–2021.72 (09/13/2021–09/17/2021; Fig. 6), coinciding with the last days of this seismic swarm. Shallow tensile sources also appear, near and in the fissure, representing the eruptive dike that used it to come to the surface. This process of magmatic intrusion in the shallow reservoir and the eruption onset ocurred in a short time period (< 6 days), as can be seen in Supplementary Fig. 3, and it is not possible separate the last stages of the pre-eruptive phase from the eruption onset sources using only InSAR data. The detection of the shallow reservoir, and even more clearly of the process indicated by the appearance of positive dip-slip sources in 2021.55 (07/20/2021), should be used as a map for the densification of the continuous GNSS network over the region, to complement the existing configuration^7^, which would have facilitated monitoring of the initial phase and the development of the eruption, studying the MPS^3,43^ and its associated fracture system in real time.

After the eruption onset, the evolution of the sources (Figs. 7–8) primarily shows direct feeding from deeper zones^30,35^ until 12/13/2021, when the eruption finishes. Nevertheless, small shallow storage forms between Jedey and Puerto Naos at about 2 km depth (Fig. 7) until the end of November. Also, some magma appears to arrive to shallow areas in the crust after the end of the eruption, but without reaching the surface (Fig. 8).

## Conclusions

We reproduce the final phase of La Palma volcanic unrest, inferring a shallow magma accumulation which begins about 3.5 months before the largest historical eruption in the island. The combination of sources obtained from this model, together with the structural crustal model, can explain the location and different characteristics of the eruption. The interpretation of the sources allows us to identifiy possibly phases of the eruption onset, but additional research, considering other eruption test cases, will be necesary to obtain more definitive conclusions.

The joint use of InSAR and GNSS deformation data coming from a dense and continuous network, combine with our interpretation methodology could help to predict the opening of possible eruptive fissures/vents during unrest episodes, potentially identifying the location, characteristics and maybe even to forecast the onset timing of an upcoming eruption. This approach would be more effective if a continuous time series inversion of ascending and descending LOS and GNSS displacements is carried out in an operative way. Similar results are expected in other active volcanic zones using this methodology of observation and interpretation in the geodetic monitoring of volcanic quiescence and unrest, and it is hoped that it will advance our understanding of volcanic activity and the forecasting of eruptions at the global scale. After sufficient testing on other eruptions of various types (fissure vs. central), it can be employed to support decision-making before eruptions, and the development of earlier response and action plans with a wider time frame. Also, it is important to note that our adjustment of the different sources implied in the final eruption processes allows us to determine, in a new, more detailed and complete way, the location and 3D geometric characteristics of the MPS and its associated fractures. It also facilitates the detection of failed eruption paths, which combined with the obtained fractures, can help to explain post-eruptive phenomena and hazards, such as the detected gases anomalies in La Bombilla and Puerto Naos^32^.

Our results demonstrate that, although this intrusion process destabilized the western flank of CV, it does not produce a collapse. Additional research in this topic is required to determine the necessary conditions for collapse and possible cascade effects (flank instability, landslide, tsunami) associated with volcanic unrest in the island.

Finally, we have shown the importance of knowing not only the MPS and crustal fracturing, but also the crustal structure, particularly in the first few kilometers below the surface, which is essential information, in combination with the recent eruptive history, for determination of the preferential zones of shallow magma accumulation and possible eruption paths. This is fundamental information in the evaluation of volcanic risk and in the planning of infrastructure and urban development in the island, and for other volcanic areas.

## Methods

### Differential Interferometric synthetic aperture radar (InSAR)

In this work, we study the deformation in La Palma using the Sentinel-1 satellite constellation. Sentinel-1 constellation uses a C-Band Synthetic Aperture Radar (SAR) sensor that, in its standard Interferometric Wide (IW) acquisition mode, provides sufficient resolution (5 m by 20 m single look) and good spatial coverage in volcanic islands^3^. In our processing we obtained a mean spatial coherence for the interferogram dataset of 0,71 in each geometry considering the entire island. The short revisit time (6 days working in the constellation), data availability and freely available historical catalogue of the satellite images make it a good choice for this study.

Sentinel-1A was launched in 2014 and the second satellite of the constellation, Sentinel-1B, in 2015, but it was not until 2017 that both satellites began to acquire images in the IW mode for the island. We considered all the available images from the two satellites of the constellation: Sentinel-1A and Sentinel-1B. To process both orbit geometries, ascending and descending, 558 Single Look Complex (SLC) Sentinel-1 Interferometric-Wide (IW) acquisitions were downloaded and processed, covering the period from January 2017 to December 2021. The radar data was provided by ESA and downloaded from the Alaska Satellite Facility (ASF)^44^. All data for each geometry were coregistered using precise orbits to a reference acquisition using ISCE software^45^ and the Network-Based Enhanced Spectral Diversity (NESD) method^46^. To remove the topographic phase from the interferograms, we used an external Digital Surface Model (DSM), derived from the high-resolution MDS05 5-m model of the Instituto Geográfico Nacional (IGN). To speed up the interferogram formation the interferogram processing was performed on a high-performance computer and the parallel software^47^ was used to distribute the work between computing nodes.

A maximum of ten connections between each date and subsequent dates were established to form twenty interferograms for each acquisition, resulting in 5470 interferograms (2755 for ascending orbit and 2715 for descending orbit). Interferograms with a temporal baseline over 300 days, perpendicular baseline over 200 m or spatial coherence below 0.6 were discarded. A multilook of 15 samples in range and 3 in azimuth was applied to the interferograms that were subsequently unwrapped using SNAPHU^48^. A summary of the used data and inteferograms generated is given in Supplementary Table 2.

To estimate the deformation time series, MintPy software was used^49^. This software uses a weighted least-squares inversion to perform the time series analysis. We choose as a reference point a high coherence point inside Tazacorte village. We selected this point as a stable zone because it is very close (~ 3 km) to the IGN’s GNSS permanent station LP02 which produced a very stable temporal series^50^ during this time period, although it is located in a low coherence area (see Supplementary Fig. 1). The atmospheric phase screen was calculated and subtracted from the deformation maps by using Global Atmospheric Model data from the European Centre for Medium-Range Weather Forecasts (ECMWF) ERA5 dataset^51^. Average velocity maps and deformation time series were obtained for each pixel with a temporal coherence^49^ over 0.8.

Comparing these results with previous findings^3^ we found three factors that have a high impact in this kind of environment: the location of the reference points, the atmospheric phase screen (APS) estimation, and the post-processing of the temporal series (filtering). The selection of the reference points (points of a known displacement rate, usually zero) can have an important impact on the results, and in small islands like La Palma can be difficult to find a good reference point that is valid over a long time span. Fernández et al.^3^ used three reference points because at that epoch those points did not appear have significant displacement, while in this work only one point, close to Tazacorte village, was used (see Supplementary Fig. 1), because the other two presented significant deformation. A map of APS for each interferogram is generated using ERA5 data and substracted from the interferogram prior the time-series analysis. Coherence Pixel Technique software (CPT), used in the previous study, also performs spatio-temporal filtering^52^ of the time series to remove remanent atmospheric artefacts that can be the result of an inaccurate atmospheric model. MintPy does not apply any post-processing of the temporal series (filtering), while in volcanic islands, such as La Palma island, as the atmosphere state decorrelates very fast, the APS is very difficult to estimate by using only the APS correction based on ERA5 model^53^. Therefore, to further deal with the residue APS pase, a spatio-temporal filtering was applied. As a result, the time series displacement provided a better result than the raw values of MintPy, and also a better agreement with the previous results^53^.

### Novelties on the inversion methodology

The modeling approach used corresponds mostly to the inversion process described by Camacho et al.^15^ but improved in several important aspects described below. In this methodology the observed ground deformation data are simultaneously modeled by deformation sources located in the subsurface domain. 3-D arbitrary sources for pressure and faults (strike-slip, dip-slip, and tensile faults from Okada formulation) are adjusted. No particular hypotheses about the nature of the source (pressure, faults), shape or location are required. The global and simultaneous data fit determines the geometrical properties and nature of the possible sources, recovering them by aggregation of small cell sources. The non-linear problem is solved by the explorative approach. The ambiguity of the problem (mostly due to the model complexity) is solved by the addition of simple regularization conditions about the total magnitude (defined as the addition of the adjusted strength values for all the adjusted sources; it describes the total departure of the model from a “non-anomalous” structure). Planar, exact or homogeneously distributed data are not required. We include the topographic effect on deformation changes by incorporating the varying-elevation analytical solution approach^15,16^.

Modeled sources are pressure changes (overpressure) and dislocation ones (tensile, dip-slip and strike-slip). In the case of pressure sources, we must consider that their existence does not necessarily imply the existence of magma acting directly in the same position. They can be related to magma sources as well as with the effect of deeper magma on hydrothermal systems or aquifers. To each of the pressure sources obtained from the inversion process an intensity value (in MPa·m^3^) is assigned, because the model equations do not allow for the separation of pressure and volume values without assuming a particular value for one of the two parameters. Given the dimensions of the study area, 25 km × 15 km, and given the quadratic attenuation of the effect of the sources of deformation with their depth, the inversion allows us to model these sources of deformation with some reliability up to a depth of about 8–10 km.

In the studied area (see Figs. 5–7) we have 24,515 pixels for ascending and 24,776 for descending LOS deformation data. Taking account the data distribution (empty zones) and taking account the estimated depths of the geodynamic phenomena to study, we carry out a subsampling of pixesl by selecting those pixels with mutual distance larger tan 280 m. It results in 1970 pixels for ascending and 2022 for descending LOS data. This subsampling allows for a much faster running and keeps geophysical information (except for the first 300 m depth).

Camacho et al.^15^ gives a complete description of the inverse approach. Here we summarize some general aspects. The inversion approach is non-linear and is based on an exploratory approach of the model space. This exploration is possible due to the use of a variable scale factor. The approach starts form a 3D grid of the whole subsurface volumen into small prismatic cells. The approach, by means of a step-by-step growth process, is successively filling selected cells with some adjusted type of deformation source and with intensity values. The misfit functions are constituted by the simultaneous fit of the data (LOS ascending and LOS descending displacement data for all the pixels) and a regularization condition based in the total size (total strength) of the resulting anomalous model. A key balance factor allows for the suitable balance between data fit and model regularity. The right selection of parameters is based into autocorrrelation analysis of the final residuals.

Camacho et al.^15^ carried out several synthetic tests to demonstrate the right performance of the proposed method. Moreover, the method was applied to real Up-Down and East–West ground deformation data coming from Mt. Etna (Sicily) and to an earthquake case. These tests cases show also the interesting application possibilities of this modeling approach. Fernández et al.^3^ applied also the former version of this methodology.

In this study we have introduced three novelties to that methodology: (1) use of Line of Sight (LOS) data from InSAR, (2) use of sequential monitoring data composed of successive data sets corresponding to successive epochs for the same site, and (3) assumption of the possible existence of offset values contained in the data sets. Now we describe these improvements in detail.

### Use of LOS data from InSAR

We assumed^15^ that deformation data consisted of values *(dx*_*i*_*, dy*_*i*_, dz_i_), *i* = *1 …np*, for deformation vector in three Cartesian components and for *np* observations points. The observation equations and the direct calculus problem (calculus of the direct effect for pressure and fault structures) were formulated for this Cartesian data.

This Cartesian approach allows for simpler expressions, but it does not correspond to the observation data configuration from InSAR. The observed satellite data essentially correspond to the radar sensor direction of the satellite (Line of Sight, LOS), one, *a*, associated to the ascending track of the satellite orbit and another, *d*, associated with the descending one. The transformation from data *a-d* to *dx-dz* data is a common practice, but it is not rigorous and can add some distortion. As a result, we modify the inversion approach to work with observation equations corresponding to LOS ascending and descending data, *a* and *d*:

1 $$\begin{aligned}&a_{i} - ac_{i} = u_{i}, \quad i = 1 \ldots na\\ & d_{j} - dc_{j} = w_{j} \quad j = 1 \ldots nd \end{aligned}$$

where *na* and *nd* represent the numbers of “ascending” and “descending” pixels (which do not necessarily coincide), and where *ac*_*i*_ and *dc*_*j*_ represent the calculated values, according to the direct problem solution, in terms of the source parameters.

Camacho et al.^15^ give the direct expression for the Cartesian calculus of values *dxc, dyc, dzc*. So, we can work and use those expressions by taking into account the relationship between the Cartesian and LOS components:

2 $$a = - dx cos\alpha_{a} \sin \beta_{a} + dy\sin \alpha_{a} \sin \beta_{a} + dz\cos \beta_{a}$$

$$d = - dx cos\alpha_{d} \sin \beta_{d} + dy\sin \alpha_{d} \sin \beta_{d} + dz\cos \beta_{d}$$

Angles *α*_*a*_*, α*_*d*_, *β*_*d*_ and *β*_*d*_ (values about 190°, − 10°, 60°, 60°) represent the heading and incidence angles for the ascending and descending LOS directions. Using these equations, we transform from the Cartesian calculated values to LOS values. From this point the approach is similar working with observation equations for LOS.

### Use of sequential monitoring

Camacho et al.^15^ considered the inversion approach for a dataset referred to a single specific epoch. However, the large availability of InSAR data provides the possibility of carrying out a temporal geodynamic monitoring of an active area, a time-series analysis. Successive deformation data sets corresponding to epochs *T*_*i*_*, i* = *1 … n* can be modeled to recover a sequential geodynamic process. For a more regular modeling of the time evolution of the active system (magmatic plumbing), we move from the irregular data epochs, *T*_*i*_*, i* = *1 … n*, to a regular sampling times *t*_*j*_, *j* = *1…m*, with a fixed sampling interval, for instance 0.1 years*.* In addition, for a more homogeneous approach, we will consider as deformation data values the deformation rates (cm/yr for instance). For each *k-th* pixel, and each sampling epoch *t*_*j*_ we obtain the value of deformation rate *a*_*jk*_*, d*_*jk*_ (cm/yr) according ascending and descending LOS using linear fitting of all previous and subsequent LOS data values *as*_*ik*_*, de*_*ik*_ (cm)*, i* = *1… n*:

3 $$\left( {\begin{array}{*{20}c} {as_{ik} } \\ {de_{ik} } \\ \end{array} } \right) - \left( {t_{j} - T_{i} } \right)\left( {\begin{array}{*{20}c} {a_{jk} } \\ {d_{jk} } \\ \end{array} } \right) - \left( {\begin{array}{*{20}c} {as_{ik} } \\ {de_{ik} } \\ \end{array} } \right)_{0} = \left( {\begin{array}{*{20}c} {u_{ij} } \\ {w_{ij} } \\ \end{array} } \right)$$

4 $$\mathop \sum \limits_{i = 1}^{n} p_{ij} \left( {u_{ij}^{2} + w_{ij}^{2} } \right) = min$$

where *u*_*ij*_ and *w*_*ij*_ are residual values, *(as*_*ik*_*)*_*0*_ and *(de*_*ik*_*)*_*0*_ are constant deformation values for LOS, and for each sampling instant *t*_*j*_ and data instant *T*_*i*_, the weight *p*_*ij*_ is determined as an exponential function of the time distance:

5 $$p_{ij} = e^{{ - \left( {\frac{{t_{j} - T_{i} }}{\tau }} \right)^{2} }}$$

The neighbor epochs have a major effect on determining the instantaneous deformation rate. This effect decreases exponentially with the temporal distance. *(as*_*ik*_*)*_*0*_, *(de*_*ik*_*)*_*0*_, *a*_*jk*_ and *d*_*jk*_ (rates) are unknowns in the former fit. *τ* is the parameter that determines the neighbor ship, and it allows for the degree of temporal continuity. After some trying we have selected the value *τ* = 0.55 years as suitable for the present study. Notice that for any time *t*_*j*_ all anterior and posterior data are used, but those with large time distance have very little influence.

System (3) is solved according to the usual least square condition (4), and we can evaluate the standard deviation *σ*_*a*_, *σ*_*d*_ of the adjustes rates *a*_*jk*_ and *d*_*jk*_. A value *τ* = 0.5 years is suitable as default for the general study. It allows to filter short wave distortions and noise and allows to keep aparently significant episodes. Nevertheless, in the present case intense deformation effects coming from the eruptive event happens quite suddenly. Then it requires to take a smaller τ value close to the beginning of the episode. A value *τ* = 0.2 − 0.3 years is suitable to get the sudden signal for the activity beginning. Conversely, in the period where there is not significant geodynamic activity a larger τ value can be useful to avoid atmospheric effects with nearly seasonal character. As a result,, we suggest carrying out a permanent test of significance of rate values *a*_*jk*_ and *d*_*jk*_ (according a significance of about four times *σ*_*a*_, *σ*_*d*_). When the rate is clearly insignificant we recalculate it with *τ* = 0.8 years. When the rate is clearly significant, we recalculate it with *τ* = 0.25 years. This approach avoids atmospheric noise for periods of no clear activity, and the detection and suitable modelling of deformation signal when geodynamic activity take place.

### Equations including offset parameters

LOS deformation data are elaborated by assuming some location (named as “seed”) with zero deformation. Then, deformation rate values *a* and *d* would be conditioned by the certainty of the seed hypothesis. In addition, the seed must have optimal interferometric qualities (coherence).

For large areas, this can be accomplished easily. However, for a small island subjected to intense geodynamic activity, as is the case at La Palma, it can be difficult to carry out. We cannot find a seed for which the ground deformation is zero with total certainty. Small deformation of the seed would appear as a nearly constant deformation component offset for the rest of the field. Across the inversion approach it would give rise to a very deep source.

Therefore, we suggest applying a process to detect and remove the global offset components of the deformation field for each epoch of the sequential approach. As detailed by Camacho et al.^15^, 2020, the inversion process for determining the deep sources corresponding to a deformation field is a “growth” process: step by step, the model grows by aggregating new cells “filled” with the source options. For each step of this growth process the provisional model (constituted by the cells aggregated in the previous steps) plus a new tentative cell (to be evaluated for suitability to aggregation) must fit the (LOS) data within a scale factor *f*.

6 $$a_{i} - ac_{i} f - oa = v_{i} \;,\;i = 1 \ldots na$$

$$d_{j} - dc_{j} f - od = w_{j} \;,\;j = 1 \ldots nd$$

where *a*_*i*_ and *d*_*j*_ are observed LOS rates (cm/yr), *ac*_*i*_ and *dc*_*j*_ are modeled or calculated rate values corresponding to the previous cells aggregation plus the new tentative cell considered to evaluate its suitability. *oa* and *od* are global offset values to be determined. *f* is the scale factor to fit the observed data. *v*_*i*_ and *w*_*i*_ are residual values. These equations include all *na* pixels for ascending LOS and all the *nd* pixels for descending LOS.

An offset would be a common fixed value that affects all the data. It cannot be adjusted by a well-defined deep source (given the extensión of the survey). For sources located within the sensitive area (below the survey area, until a depth about a half of the survey diameter) the method adjusts a source instead of an offset value. Below the sensitive area the methods tends to adjust a no-null offset.

The inversion approach considers observation Eqs. (6) plus some additional regularization equations to avoid the non-uniqueness problems and guarantee the filtering of the uncorrelated data noise. The regularization conditions can be written for residuals^15^ as:

7 $$S = {\varvec{v}}^{T} {\varvec{P}}1\user2{ v} + {\varvec{w}}^{T} {\varvec{P}}2\user2{ w} + \lambda f{\varvec{m}}^{T} \user2{Q m} = min$$

***v*** and ***w*** are the vectors for residuals. ***m*** is a vector for the magnitude of the anomalous model (evaluated as the volume of cells multiplied by the deformation strength of the cells). ***P****1*, ***P****2* and ***Q*** are suitable cofactor matrices^15^. Finally, *λ* is a balance factor that regulates the geometrical smoothness of the model and corresponds to the balance between data fit (residuals *v* and *w*) and model size (*m*). *S* is the misfit function. System (6) and (7) can be solved for parameters *f*, *oa* and *od*. By substituting (6) into (7) we get:

8 $$\begin{aligned} & S_{a a} - 2 oa S_{a u1} - 2 f S_{a ac} + oa^{2} S_{u1 u1} + 2 oa fS_{ac u1} + f^{2} S_{ac ac} + S_{d d} - 2 od S_{d u1} \\ & \quad - 2 f S_{d dc} + od^{2} S_{u2 u2} + 2 od fS_{dc u2} + f^{2} S_{dc dc} + \lambda f^{2} S_{mm} = min \\ \end{aligned}$$

where for instance,

9 $$S_{a ac} = {\varvec{a}}^{T} {\varvec{P}}1\user2{ac}, S_{a u} = {\varvec{a}}^{T} {\varvec{P}}1\user2{u}, S_{ac u} = {\varvec{ac}}^{T} {\varvec{P}}1\user2{u}, S_{mm} = {\varvec{m}}^{T} {\varvec{Qm}}$$

with *u*_*i*_ = *(0 0 1*_*i*_* 0 0)*.

The minimization condition (7) with respect to *f*, *oa* and *od* brings to the following derivate equations:

10 $$\frac{d}{d oa}S = 0, \frac{d}{d od}S = 0, \frac{d}{d f}S = 0$$

Developing (10), (8) and (9), we get:

$$d1 S_{u1u1} + f S_{ac u1} - S_{a u1} = 0$$

11 $$d2 S_{u2u2} + f S_{dc u2} - S_{d u2} = 0$$

$$d1 S_{ac u1} + d2 S_{dc u2} + f \left( {S_{ac ac} + S_{dc dc} + \lambda S_{mm} } \right) - (S_{a ac} + S_{d dc} ) = 0$$

Adjusted values can be written as:

$$f = \frac{{(S_{a ac} + S_{d dc} ) - oa S_{ac u1} - od S_{dc u2} }}{{(S_{ac ac} + S_{dc dc} + \lambda S_{mm} )}}$$

$$oa = \frac{{S_{a u1} (S_{ac ac} + S_{dc dc} + \lambda S_{mm} ) - S_{ac u1} \left( {S_{a ac} + S_{d dc} } \right) + d2 S_{ac u1} S_{dc u2} )}}{{S_{u1 u1} (S_{ac ac} + S_{dc dc} + \lambda S_{mm} ) - S_{ac u1} S_{dc u2} }}$$

$$od = \frac{{S_{d u2} (S_{ac ac1} + S_{dc dc} + \lambda S_{mm} ) - S_{dc u2} \left( {S_{a ac} + S_{d dc} } \right) + d1 S_{ac u1} S_{dc u2} )}}{{S_{u2 u2} (S_{ac ac} + S_{dc dc} + \lambda S_{mm} ) - S_{dc u2} S_{dc u2} }}$$

Once *f*, *oa* and *od* (scale factor and offset values for ascending and descending LOS) have been calculated, by substituting in (7) we determine the misfit value *S* for the tentative cell (and its tentative parameters: nature, angles, sign, etc.) and then we evaluate its suitability. That tentative cell (and parameter values) that produce a minimum value for *S* is selected to be definitively aggregated to the growing model for the 3D source structure. The adjusted offset values produce, step by step, the best fit for (6), (7), and (8) and then the more suitable source model.

## Computer code

Defsour® software for inversion of LOS data, which runs under Windows 10 operative system, is available under request to the authors using a transfer agreement, for volcano monitoring and research, excluding commercial applications.

## Supplementary Information

Supplementary Information.

Supplementary Movie S1.

Supplementary Movie S2.

Supplementary Movie S3.

Supplementary Movie S4.

Supplementary Movie S5.

Supplementary Movie S6.

## Data Availability

SLC data from Sentinel-1 is publicly available on the Alaska Satellite Facility website: www.asf.alaska.edu.
