# Supplementary material for: Shallow magmatic intrusion evolution below La Palma before and during the 2021 eruption
Source: Sci Rep. 2022 Dec 12;12:20257. doi: 10.1038/s41598-022-23998-w (PMC9744821; doi:10.1038/s41598-022-23998-w)
Supplement: Supplementary file 1 — Supplementary Information. [file 41598_2022_23998_MOESM1_ESM.pdf]

Supplementary Information for

Shallow magmatic intrusion evolution below La Palma before and during the 2021 eruption

José Fernández[1,*,+], Joaquin Escayo[1], Antonio G. Camacho[1,+], Mimmo Palano[2], Juan F. Prieto[3], Zhongbo Hu[4,8,++], Sergey V. Samsonov[5], Kristy F. Tiampo[6], Eumenio Ancochea[7]

[1]Instituto de Geociencias (CSIC, UCM). Calle del Doctor Severo Ochoa, nº 7. Ciudad Universitaria. 28040-Madrid, Spain.

[2]Istituto Nazionale di Geofisica e Vulcanologia, Osservatorio Etneo - Sezione di Catania, Piazza Roma 2, 95125 Catania, Italy.

[3]ETS de Ingenieros en Topografía, Geodesia y Cartografía, Universidad Politécnica de Madrid, 28031-Madrid, Spain.

[4]Dares Technology, C/ Esteve Terrades, 1, Building RDIT Office 117, Parc UPC – PMT 08860, Castelldefels Barcelona, Spain.

[5]Canada Centre for Mapping and Earth Observation, Natural Resources Canada, 560 Rochester Street, ON K1A 0E4, Ottawa, Canada.

[6]Cooperative Institute for Research in Environmental Sciences (CIRES), 216UCB, University of Colorado at Boulder, Boulder, CO, 80309, USA.

[7]Departamento de Mineralogía y Petrología, Fac. CC. Geológicas, Universidad Complutense de Madrid, 28040 Madrid, Spain.

[8]Also at CommSensLab, Dep. Signal Theory and Communications, Universitat Politècnica de Catalunya (UPC), D3-Campus Nord-UPC, C. Jordi Girona 1-3, 08034, Barcelona, Spain.

*Correspondence to: jft@mat.ucm.es

[+]These authors contributed equally: José Fernández, Antonio G. Camacho

[++]Now at: Instituto de Geociencias (CSIC, UCM). Calle del Doctor Severo Ochoa, nº 7. Ciudad Universitaria. 28040-Madrid, Spain.

This file includes:

Supplementary Text
Supplementary Figures. 1 to 9
Supplementary Tables 1 to 2
Supplementary References
Captions for Supplementary Movies 1 to 6

Other Supplementary Materials for this manuscript include the following:

Supplementary Movies 1 to 6

Supplementary Text

Full details of the structural gravimetric modeling

Important and useful information for framing both discussion and interpretations of the origin of the deformations detected in volcanic areas is the comparison with the knowledge of the crustal structure of the active zone[1]. Valuable crustal structure information for La Palma Island comes from the modeling of gravity data modelling by using a fitted three-dimensional (3D) model for anomalous bedrock densities as we do for La Palma Island. This model reflects clear morphological patterns that help explain local volcanic and geodynamic behavior.

The gravimetric data were recorded in 2006 and corresponded to 314 stations[2], which are distributed throughout the island (except in some areas of very difficult access). Our gravimetric data for the structural anomaly study were obtained on mutually separated points at distances greater than 1.2 km covering the full island. For these distances, the resulting anomaly, as is usual in this type of study, has accuracies on the order of 0.5-1.0 mGal, and the possible effects produced by volcanic activity are normally significantly lower than this precision threshold. Therefore, the possible geodynamic variations of gravity have a negligible effect in large-scale (the whole island) structural studies. The gravimetric anomaly values corrected for topographic effects show a strong gradient (125 mGal) between the centre-north of the island and the southern tip[2]. Camacho et al.[2] used these gravimetric data to develop a three-dimensional (3D) model of anomalous density structures on La Palma using an almost automatic inversion process. This model assumes that the density anomalous bodies are embeddes in a homogeneous medium, and that, in addition to fitting the observed data, they verify the usual conditions of regularity (i.e minimizing of the total anomalous mass of the resulting source model in addition to data fit).

Recent work[3,4] improved the inversion methodology by also considering stratified structures, with increasing density with depth. Using this approach, somewhat more realistic anomalous models are obtained. This new approach was used in the modeling of all available gravity data at La Palma[5]. The main feature of that model is the existence and morphology of a very large and dense intrusive body located below the Northern Volcanic Complex (NVC)

corresponding to dike swarms and accumulated plutonic material. This outstanding structure is responsible for a gravimetric anomaly variation of about 130 mGal in 30 km, from its centre under the Taburiente caldera to the southern end of the island.

Considering that this large anomaly associated with the NVC of the Island can mask or distort other anomalies of smaller magnitude or extent existing on the island, we decided to test the option of using only the data from the southern part of the island (so that the main part of the NVC anomaly is excluded). To do this, we have applied the same gravimetric inversion methodology to the data reflected in Supplementary Fig. 4.

The inversion process starts by dividing the volume of the subsoil into a partition of small prismatic cells. In this case, we have considered approximately 90000 cells with an average size of 300 m that cover the subsoil to a depth of 9000 m with sufficient resolution. Furthermore, we have considered a regular layering in sub-horizontal layers (12 layers, arbitrarily, trying to offer information but avoiding very realistic models which could be represented by a model of few layers) of increasing density in depth as initial configuration to be modified in the inversion process. Finally, we have chosen an adequate value of the regularization parameter capable of separating correlated gravity signal from non-correlated noise. For that we analyze the resulting auto-correlation of the final residuals. The regularization value is chosen as that producing a null auto-correlation of residuals[2]. Once these parameters are set, the inversion process is automatic and a 3D model of anomalous densities is obtained.

Figs. 3 and 4 shows some results of the adjusted 3D density model. Looking at these figures, the following structural features can be seen:

a) The presence of the important Northern Volcanic Complex, NVC (Fig. 3), which dominates the centre and north of the island, stands mostly out in all of them. It is consistent with the previous results[2,5–7], including the updated ones for the full Island[5].

b) A smaller positive anomaly also is detected to the south, which was hidden in the results for the complete Island. It suggests the presence of an incipient Southern Volcanic Complex, SVC (Fig. 3), located outside the island to the southwest of it. As Camacho et al.[2] noted, a clear local minimum in the regional Bouguer disturbance is observed close to the SE border of La Palma (see Supplementary Figure 6 in the Supplementary Information from Fernández et al.[5]), which should correspond to the thickness pattern of the crust in this area. This was associated

with the presence of a thermal anomaly corresponding to recent volcanism, as the Teneguía volcano eruption which took place in this area in 1971, as also suggested by the long alignment of seamounts inferred from the bathymetric data southward to La Palma[2,8]. All those previous results agree with, and support, our findings.

c) The results, up to 1000-1500 m below sea level (bsl) are very similar, for both cases, the obtained for the entire island (see Figs. 3-4 and Fig. 6 and Supplementary Fig. 7 by Fernández et al.[5]), and our results for approximately the southern half, except for the extreme south. Discrepancies from other previous results[2,5] below these depths come from the different methodologies used for the inversion of the gravimetric data. We infer that the alignments of minimums reflect fractured surface areas associated with the N-S rift structure in CV[2,5]. These low-density structures, mainly on the western flank of the ridge, are formed by several small in echelon tracks with directions not far from that of the SW side of the main body[2,5]. Our updated results still delineating, at shallow level (Figs. 3-4), the alignment of low-density small bodies in the summit line, corresponding to the alignment of the last eruptive activity on the island. Those shallow (close to sea level) alignments of minima in the former figures (and our updated results) can be associated[2,5–7] with zones of shallow fractures associable to the N–S rift structure in CV. In Figs. 3-4, a low-density structure is detected beneath the western slopes of CV[2,5–7]. It is suggested that this body might represent either debris-avalanche and hyaloclastite deposits from CN collapse or significant deposits of an older sedimentary apron that surrounds the high-density core, or some combination of the two. The significant thicknesses may be the result of the superposition of long-term denudation of subaerial material or old collapses from the northern shield volcanoes. E-W profiles seem to indicate that extension diminishes southwards with a relatively larger thickness in the northern part of CV and smaller amount of material at the southern part of the island. Magnetotelluric survey[7,9,10] suggests the existence at similar depths of a low-resistivity body, which also confirms that this hypothesis is correct and the CV volcano series rests on the remains of the collapsed CN ridge (a breccoid layer). This low-density body also is spatially coincident in its depth range with the modelled fault planes obtained by González et al.[7] from InSAR displacements they obtained from ERS-1,2 and ENVISAT images in the times periods 1993-2004, 2006-2008 (see their Figure 10). Those results also are consistent with the lower bounds of possible intravolcanic structures, such as the CN collapse scar (debris avalanche process), or the very upper depth limits for the contact between pre-volcanic sediments and island-

volcanic slumping process[7]. This area also is related to the hydrothermal system[3,10] around CV.

d) A clear extension of the NVC towards the southeast (Northern Volcanic Complex Extension, NVCE), partially below and towards CV, is delineated. We also observe a possible northeast extension of the SVC, the Southern Volcanic Complex Extension (SVCE), going again towards CV, arriving below the summits. See Figs. 3-4.

e) As in the case for other volcanic Islands, NVC would correspond to large magma supply from the mantle, resulting in a complex edifice and volcanic structure formed by swarms of dikes mostly with radial symmetry. SVC also could be an incipient volcanic complex. These intrusive large bodies are normally denser than the surrounding volcanic rock in which they fit, so positive gravity anomalies indicate preferred areas of magma ascent throughout the volcanic complex.

f) We interpret the new findings (Figs. 3-4), NVCE and SVCE, as composed of thousands of narrow dikes or other sheet-like intrusions propagated laterally from volcanic centres, NVC and SVC, along rift zones, following neutral buoyancy levels. They would represent sub-horizontal (and ascending paths, about 4-5 km depth), without deep roots for magma supply below. After the subsequent cooling and compaction of the magmatic material, these lateral structures would show positive anomalies of relative density concerning their environment (and concerning the materials immediately below them). They may suffer from some gravitational instability, with a tendency to subside in the very long term, much longer than the time period studied here with InSAR. This depth would correspond to the shallowest level of magma stagnation before the final eruptive phase[5,11–15] with short-term storage or an intrusive complex within the volcanic edifice. Similar depth for shallow magma stagnation appears in other islands of the Canaries (e.g., El Hierro[16]; Gran Canaria[13]; Tenerife[17]) and may represent a regional horizon of neutral buoyancy for the Canary Islands magma[12]. It corresponds to the average pre-island seafloor with a variable effect due to lithospheric flexural volcanic loading[16].

g) We observe a possible fracture alignment (Fig. 4) connecting the centres of the NVC and SVC, and another one delimiting the abrupt SE and SW edges of the NVC. González et al.[7] consider that the discontinuity in the border of the high-density body could represent a contrast between different materials, as expected in the buried "Coebra" collapse structure.

h) The structural evolution to the south should be consistent with the migration of the volcanism in La Palma in this direction[11]. Our result agrees with Camacho et al.[2] that pointed out that the southward elongation of the NVC suggests a possible change of the magmatic pattern toward the volcanism of CV complex. Previous works[7,18] conclude that TC-CN and CV volcanic systems are not only spatially separated but also represent two distinct volcanoes with separate magma plumbing systems.

Extended technical description of the Modeled Sources

We performed a checker-board resolution test to ensure that InSAR results are sufficient for the inversion. We computed ascending and descending LOS for the modeled sources using the modeled sources for each kind of source described in the Methods section, and compare the obtained values with the observed ones (see Supplementary Fig. 5). The data fit is quite good for both data sets, and the root-mean square of the residuals (observed-modeled LOS) is of: about 1 cm/yr for periods before and after the jump and about 3.5 cm/yr for the jump values (2021.71). This is a good estimation of the uncorrelated noise level present in the LOS ascending and descending data deformation rates in the selected epoch t_i. The displacement data show several epochs with the clear systematic signal of deformation over the noise background. These signals correspond to values with non-null autocorrelation and they are the data we use for inversion.

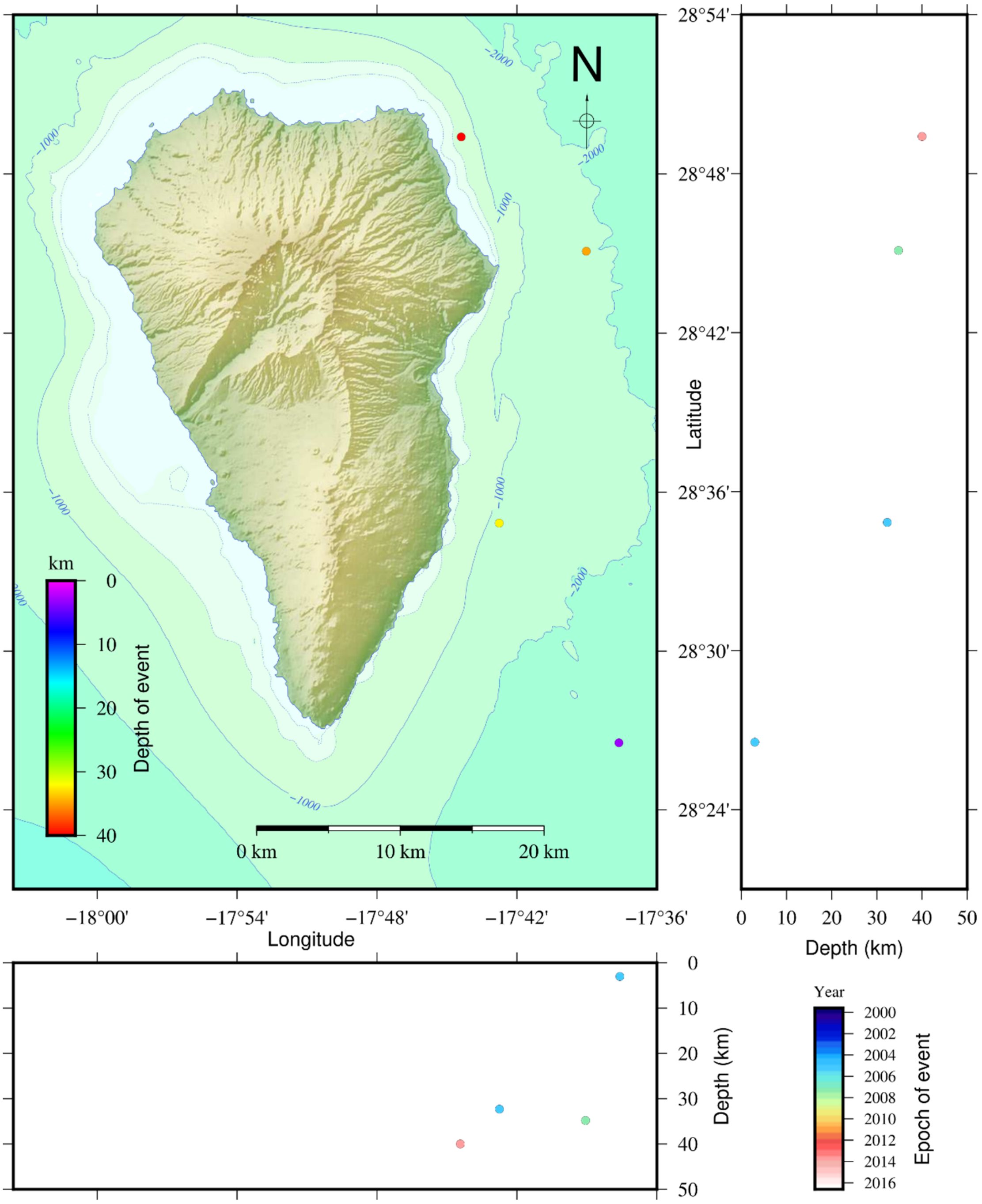

Supplementary Fig. 1. Seismicity under La Palma island during 2000-2016. Graphic representation of the detected seismicity[19] during the period January 2000-December 2016. GMT software (www.generic-maping-tools.org) was used to create this figure.

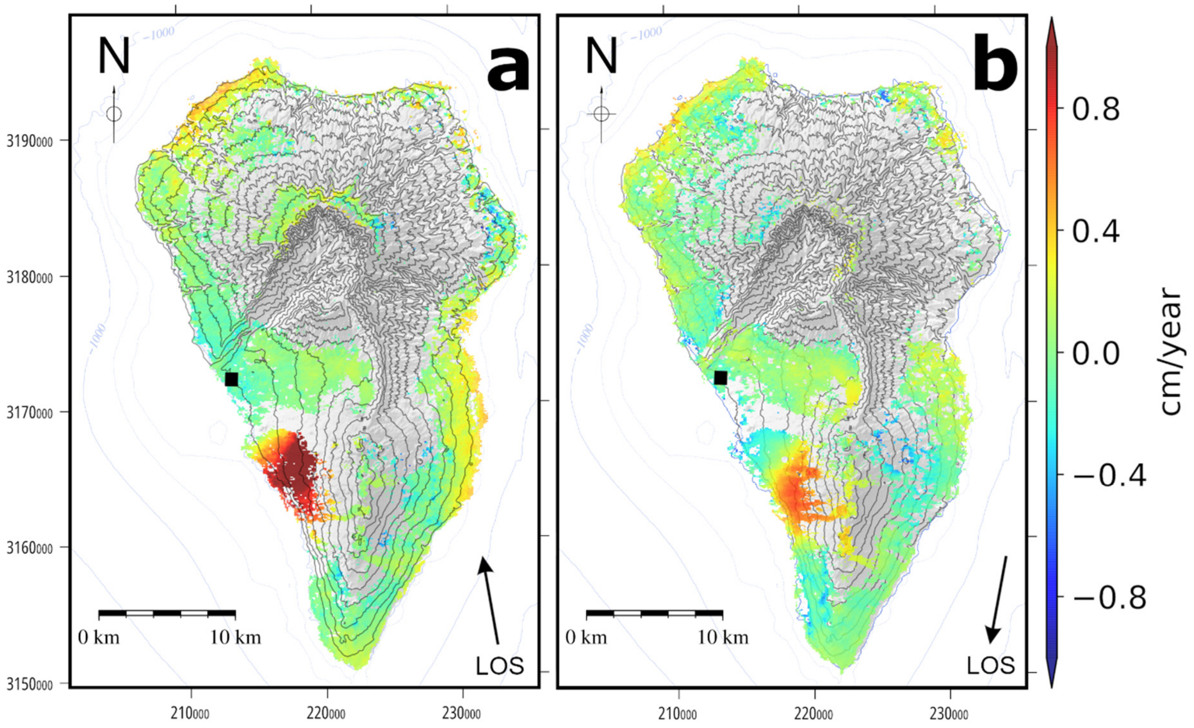

Supplementary Fig. 2. InSAR mean velocity maps. LOS InSAR mean velocity maps for ascending and descending geometries for the 2017-2021 period. The dark square identifies the reference point in Tazacorte village. a) ascending results, b) descending results. UTM28 North reference system. Coordinates in m. GMT software (www.generic-maping-tools.org) was used to create this figure.

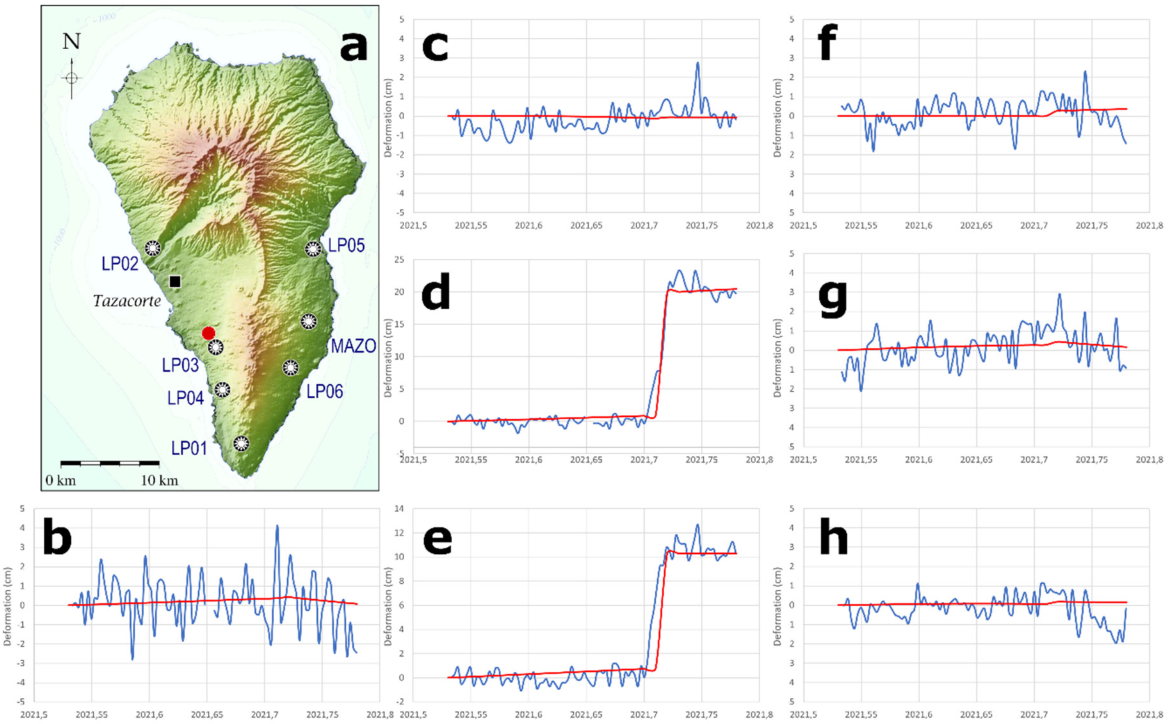

Supplementary Fig. 3. Comparison between InSAR results and GNSS permanent stations. Comparison between GNSS data from IGN's permanent stations[19,20] (blue lines) with the InSAR smoothed deformation data (red lines). For the comparison the GNSS data has been projected into the LOS vector and compared with the closest pixel used for the data inversion. InSAR data of LP01, LP02, LP05 and LP06 stations were selected from an ascending orbit while LP02, LP04 and MAZO stations are from a descending orbit and red dot illustrates the location of the pixel used to show the temporal series in Fig. 2 of the main text. **a**) Location of the IGN's permanent stations. Black square represents the area used as reference. Panels **b**) to **g**) LP01 to LP06 stations, **h**) MAZO station. GMT software (www.generic-mapping-tools.org) and Microsoft Excel 2016 were used to create this figure.

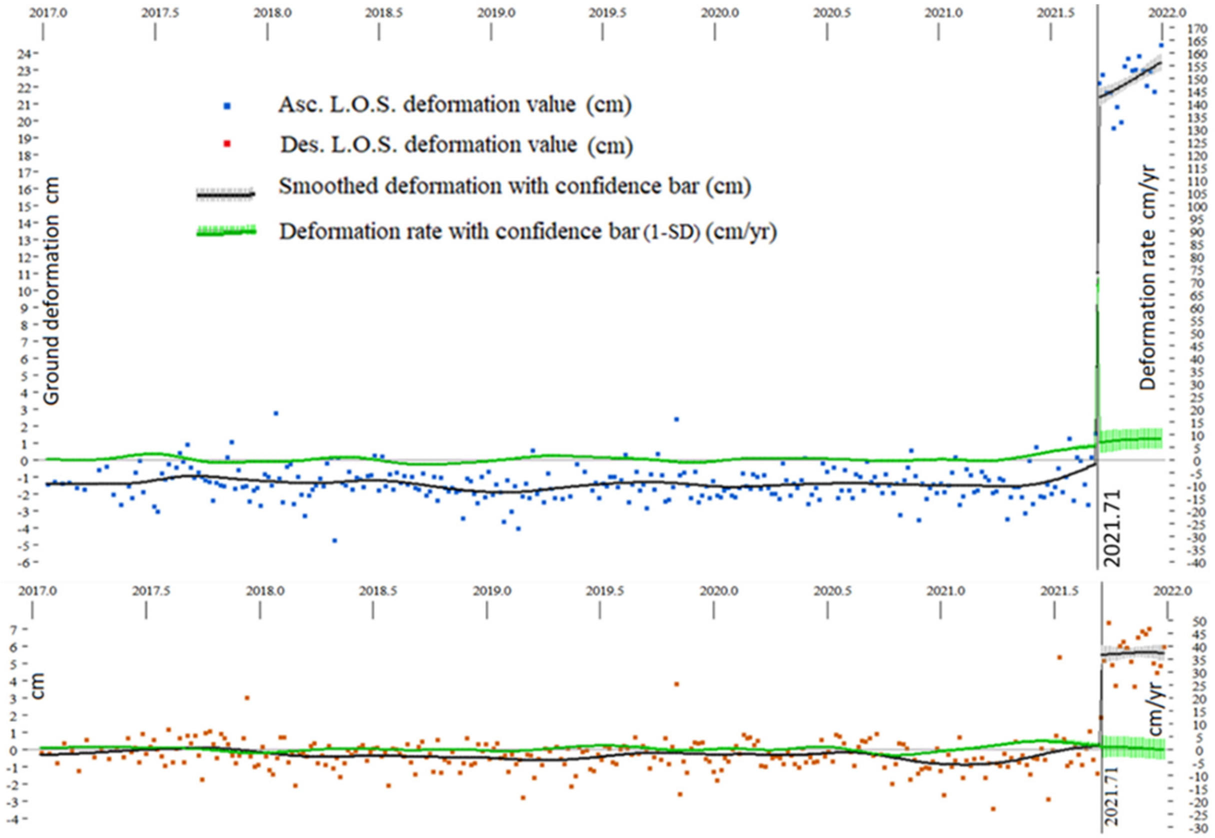

Supplementary Fig. 4. Input deformation data for a pixel close to the maximum deformation site with UTM coordinates (218000,3166000). Dots denote the discrete satellite data (blue for ascending and red for descending LOS displacements). Black lines are smoothed deformation (cm) (left axis) with confidence bars for one standard deviation. Smoothing approach is described in the Methods section. Green lines are smoothed deformation rate (cm/yr) (right axis) with confidence bars. At 2021.71 (09/17/2021) there is a clear jump in the deformation record. See location of the pixel in Supplementary Fig. 2. Matlab software (www.mathworks.com) was used to create this figure.

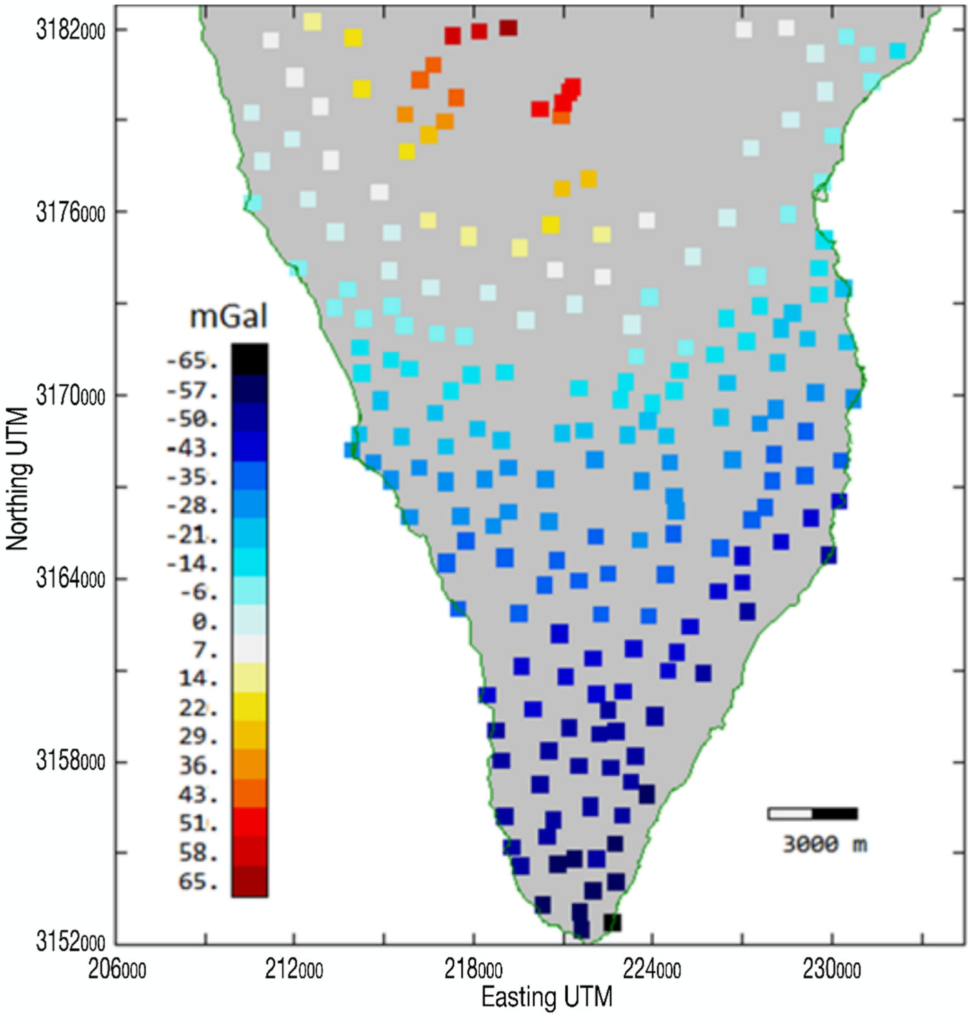

Supplementary Fig. 5. Gravity data used to model the crustal structure of La Palma. Location of the 194 gravimetric stations in the studied south part of La Palma Island, and values of relative gravimetric anomaly (step 5 mGal) corrected for topography. UTM28 North reference system. Coordinates in m. Matlab (www.mathworks.com) and GMT (www.generic-maping-tools.org) softwares were used to create this figure.

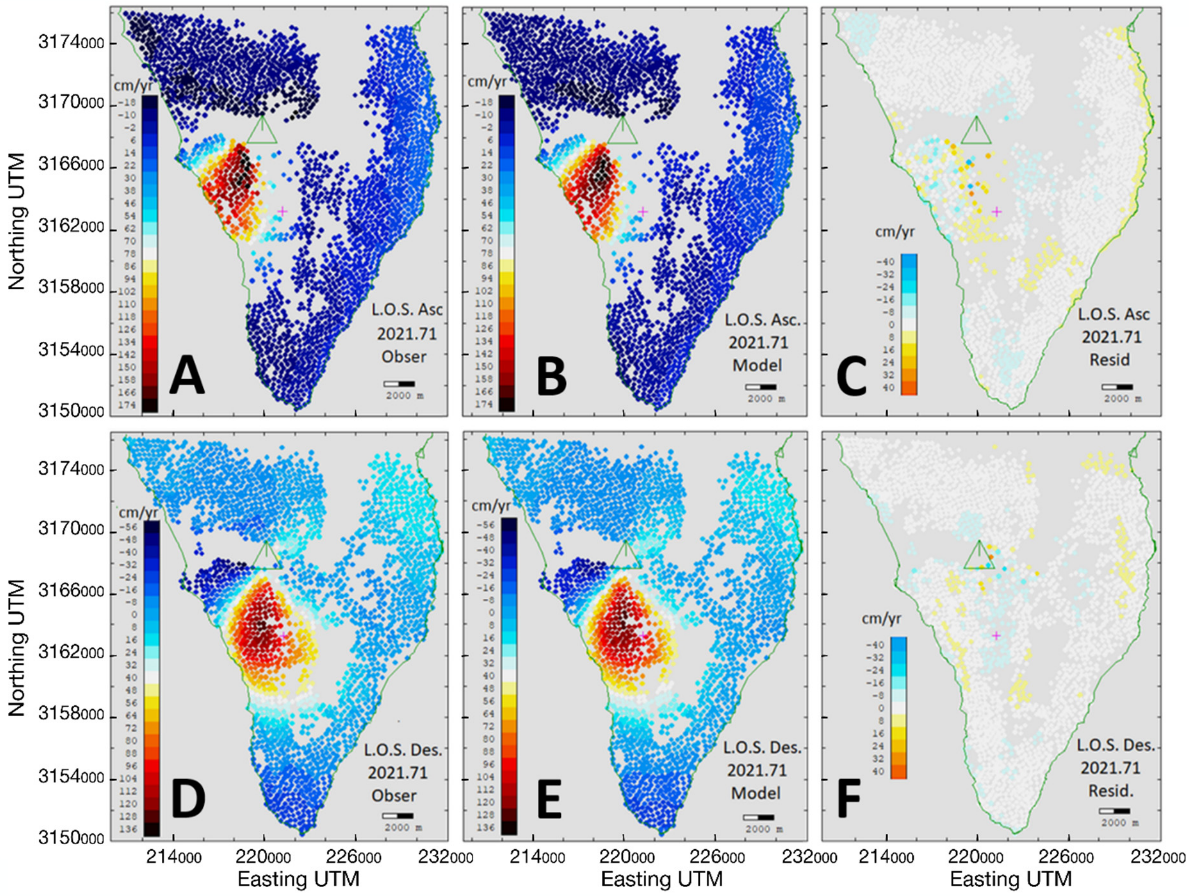

Supplementary Fig. 6. Checkerboard example. Epoch 2021.71 (09/17/2021). Input data (**A, D**), modelled values (**B, E**) and residual values (**C, F**) for the deformation rate (cm/yr) for the LOS. ascending (**A-C**) and descending (**D-F**) displacement rates. For this epoch, deformation rates are very high (e.g., about 180 cm/yr for ascending data.). The fit is good, and final residuals for 2021.71 are about 4 and 3 cm/yr r.m.s for ascending and descending data respectively. UTM28 North reference system. Coordinates in m. Matlab (www.mathworks.com) and GMT (www.generic-maping-tools.org) softwares were used to create this figure.

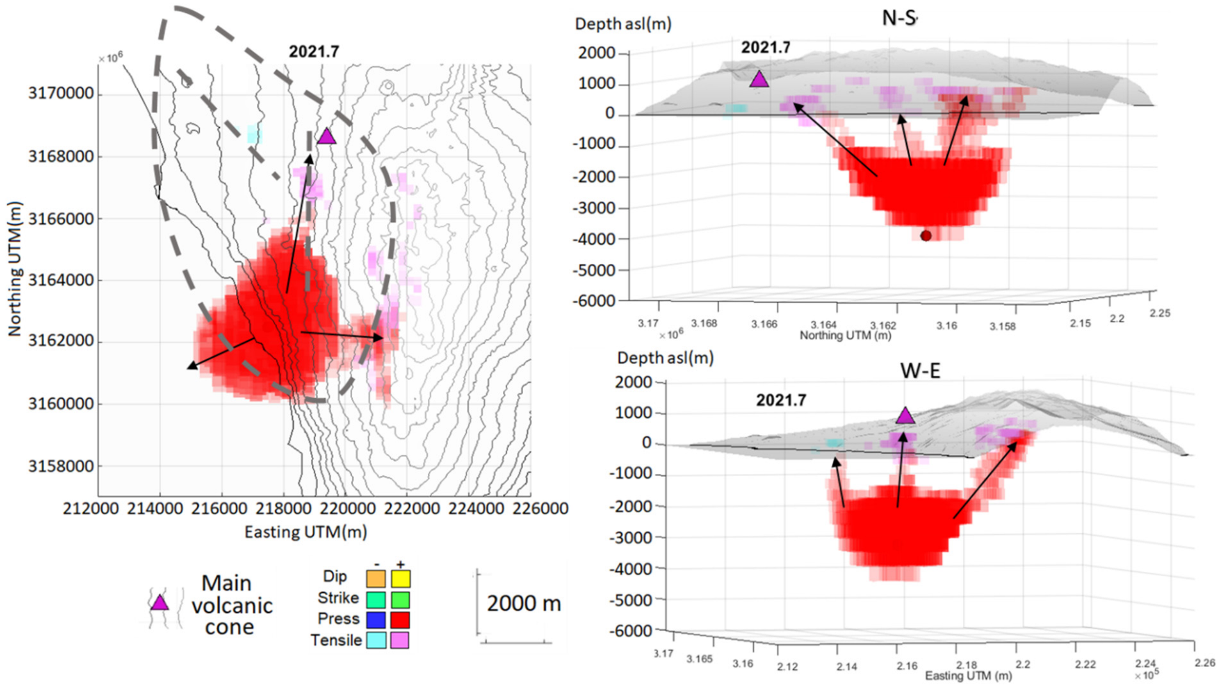

Supplementary Fig. 7. Time evolution of the modeled pressure and tensile sources for 09/13/2021-09/20/2021 (2021.70-2021.72) period. Horizontal and vertical N-S and W-E projections of the pressure and tensile sources obtained for the jump in displacements measured in 2021.70-2021.72. The horizontal projection has superimposed the outline of the low-density area and alignments as shown in Fig. 4. Looking at the three branches in the represented 3D source we can see the tensile sources in their upper part (representing dykes, what they should conceptually be). UTM28 North reference system. Coordinates in m. Matlab (www.mathworks.com) and GMT (www.generic-mapin-tools.org) softwares and Microsoft PowerPoint 2016 were used to create this figure.

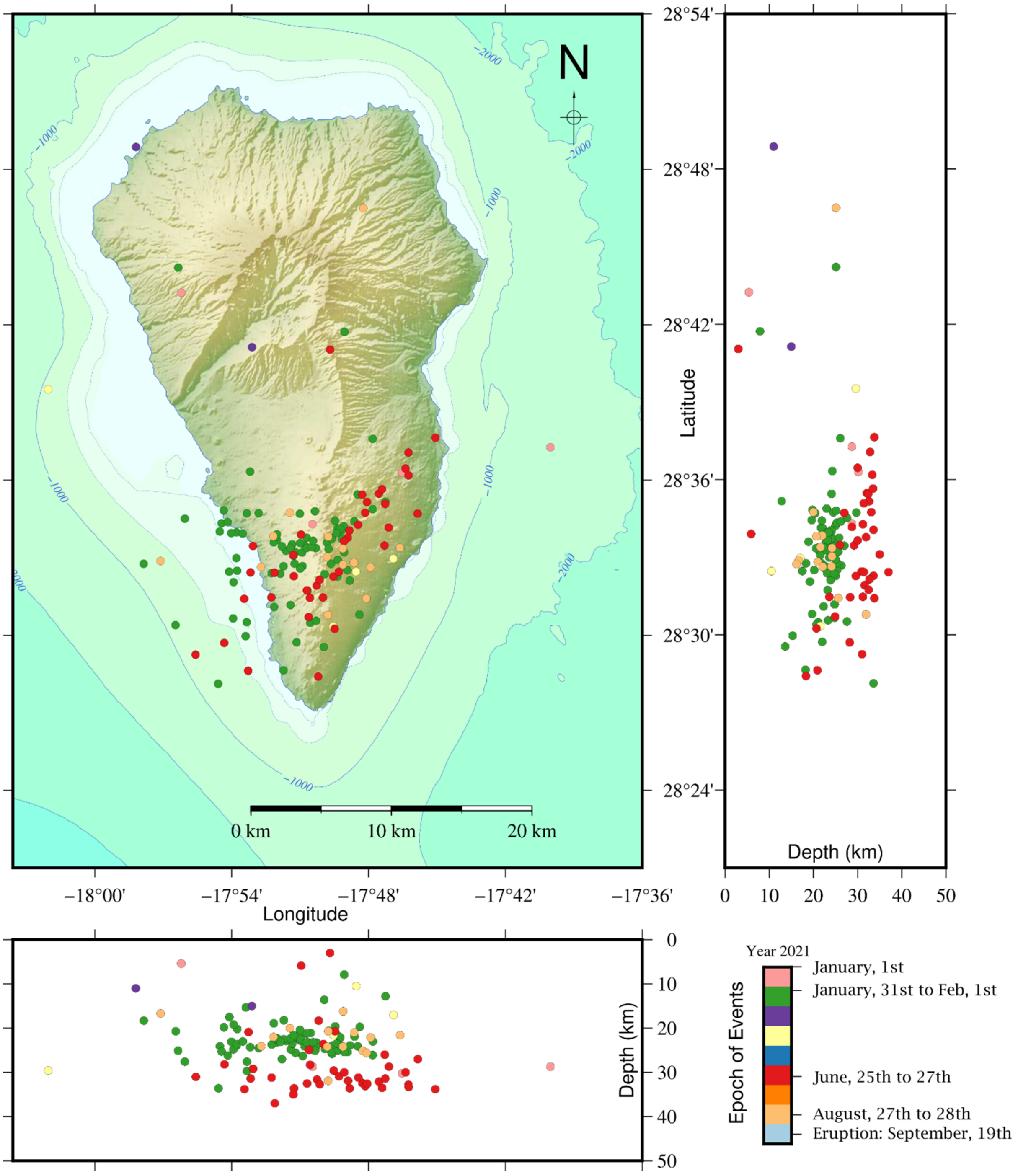

Suplementary Fig. 8. Seismic swarms under La Palma island during January-August 2021. Graphic representation of the detected[19] seismic event detected and included in the seismic swarms occurred during the period January 2021-August 2021. GMT software (www.generic-maping-tools.org) was used to create this figure.

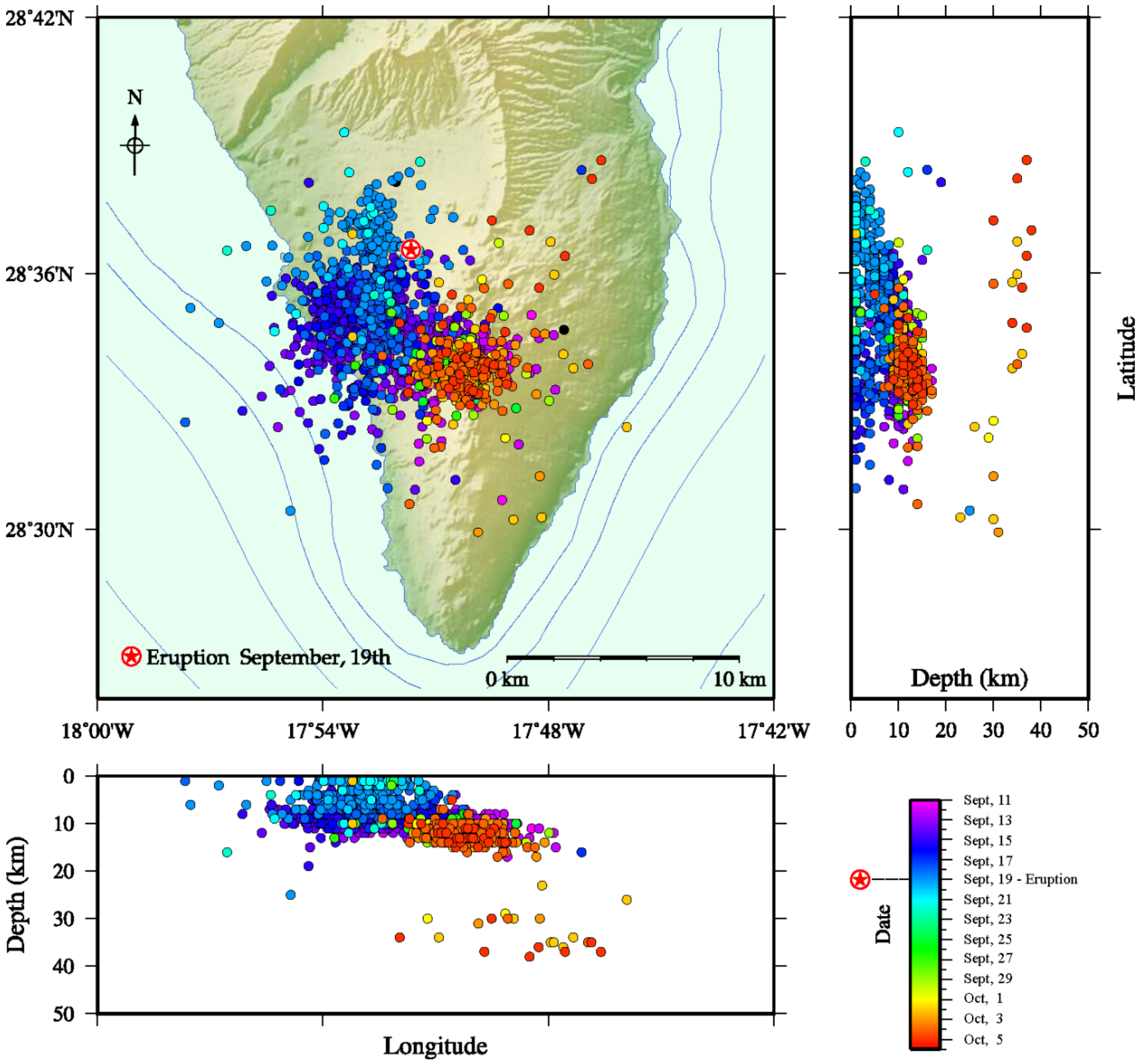

Suplementary Fig. 9. Seismic swarm under La Palma island during September 11-October 5, 2021. Graphic representation of the detected seismic events[19] included in the seismic swarm during September, 11-October, 5 2021, with accompanied the eruption onset. GMT software (www.generic-maping-tools.org) was used to create this figure.

Supplementary Table 1. Seismic swarms occurred beneath La Palma island from October 2017 to the date of the eruption. The table includes the number of seismic events detected and the events finally located in each swarm[19], when this information is available.

SEISMIC SWARM	DATES	SEISMIC EVENTS
1	7-13/10/2017	352 detected, 121 localized
2	10-15/02/2018	928 detected, 84 localized
3	24/07-02/08/2020	682detected, 160 localized
4	8-18/10/2020	69 localized
5	21/11/2020	118 detected, 14 localized
6	23-26/12/2020	602 detected, 130 localized
7	31/01-01/02/2021	77 localized
8	25-27/06/2021	37 localized
9	11-19/09/2021	6632 detected, 1373 localized. Eruption starts on Sept, 19th.

Supplementary Table 2. Summary of the dataset's main parameters. All available SLCs were included in this study for the considered time period.

	Ascending	*Descending*
Start date	20170120	20170128
End date	20211231	20211227
Number of SLC	277	281
Track / Relative orbit	60	110
Azimuth (°)	349.6	190.0
Mean Incidence (°)	39.21	39.23
Interferograms generated	2715	2805
Interferograms discarted	2478	2533

References

1. Fernández, J., Pepe, A., Poland, M. P. & Sigmundsson, F. Volcano Geodesy: Recent developments and future challenges. *J Volcanol Geotherm Res* **344**, 1–12 (2017).
2. Camacho, A. G. *et al.* Structural results for La Palma island using 3-D gravity inversion. *J Geophys Res* **114**, B05411 (2009).
3. Camacho, A. G., Fernández, J. & Gottsmann, J. A new gravity inversion method for multiple subhorizontal discontinuity interfaces and shallow basins. *J Geophys Res* **116**, B02413 (2011).
4. Camacho, A. G., Prieto, J. F., Aparicio, A., Ancochea, E. & Fernández, J. Upgraded GROWTH 3.0 software for structural gravity inversion and application to El Hierro (Canary Islands). *Comput Geosci* **150**, 104720 (2021).
5. Fernández, J. *et al.* Detection of volcanic unrest onset in La Palma, Canary Islands, evolution and implications. *Sci Rep* **11**, 2540 (2021).
6. Prieto, J. F. *et al.* Geodetic and Structural Research in La Palma, Canary Islands, Spain: 1992–2007 Results. *Pure Appl Geophys* **166**, 1461–1484 (2009).
7. González, P. J., Tiampo, K. F., Camacho, A. G. & Fernández, J. Shallow flank deformation at Cumbre Vieja volcano (Canary Islands): Implications on the stability of steep-sided volcano flanks at oceanic islands. *Earth Planet Sci Lett* **297**, 545–557 (2010).
8. Sandwell, D. T. & Smith, W. H. F. Marine gravity anomaly from Geosat and ERS 1 satellite altimetry. *J Geophys Res Solid Earth* **102**, 10039–10054 (1997).
9. Garcia, X. & Jones, A. G. Internal structure of the western flank of the Cumbre Vieja volcano, La Palma, Canary Islands, from land magnetotelluric imaging. *J Geophys Res* **115**, B07104 (2010).
10. di Paolo, F. *et al.* La Palma island (Spain) geothermal system revealed by 3D magnetotelluric data inversion. *Sci Rep* **10**, 18181 (2020).
11. Klügel, A., Galipp, K., Hoernle, K., Hauff, F. & Groom, S. Geochemical and Volcanological Evolution of La Palma, Canary Islands. *J Petrol* **58**, 1227–1248 (2017).
12. Lodge, A., Nippress, S. E. J., Rietbrock, A., García-Yeguas, A. & Ibáñez, J. M. Evidence for magmatic underplating and partial melt beneath the Canary Islands derived using teleseismic receiver functions. *Phys Earth Planet Inter* **212–213**, 44–54 (2012).
13. Romero Ortiz, J. La Erupción del Nambroque en la Isla de La Palma. *Bol Inst Geol Min Esp* **63**, (1951).
14. Hansteen, T. H., Klügel, A. & Schmincke, H.-U. Multi-stage magma ascent beneath the Canary Islands: evidence from fluid inclusions. *Contrib Mineral Petrol* **132**, 48–64 (1998).
15. Klügel, A., Hansteen, T. H. & Galipp, K. Magma storage and underplating beneath Cumbre Vieja volcano, La Palma (Canary Islands). *Earth Planet Sci Lett* **236**, 211–226 (2005).
16. González, P. J. *et al.* Magma storage and migration associated with the 2011-2012 El Hierro eruption: Implications for crustal magmatic systems at oceanic island volcanoes. *J Geophys Res Solid Earth* **118**, 4361–4377 (2013).

17. Andújar, J., Costa, F. & Martí, J. Magma storage conditions of the last eruption of Teide volcano (Canary Islands, Spain). *Bull Volcanol* **72**, 381–395 (2010).
18. Galipp, K., Klügel, A. & Hansteen, T. H. Changing depths of magma fractionation and stagnation during the evolution of an oceanic island volcano: La Palma (Canary Islands). *J Volcanol Geotherm Res* **155**, 285–306 (2006).
19. Instituto Geográfico Nacional. Volcanic activity surveillance. https://www.ign.es/web/ign/portal/vlc-area-volcanologia.
20. de Luca, C. *et al.* Pre- and Co-Eruptive Analysis of the September 2021 Eruption at Cumbre Vieja Volcano (La Palma, Canary Islands) Through DInSAR Measurements and Analytical Modeling. *Geophys Res Lett* **49**, e2021GL097293 (2022).

Title: Supplementary Movie 1.

Description: La Palma Island. Volcano area. Plan projected views. Sequential plan projected views of the adjusted models (2017.0-2022.0) for (incremental) deformation sources obtained from ascending and descending LOS radar data.

Title: Supplementary Movie 2.

Description: La Palma Island. Volcano area. SN projected views. Sequential SN projected views of the adjusted models (2017.0-2022.0) for (incremental) deformation sources obtained from ascending and descending LOS radar data.

Title: Supplementary Movie 3.

Description: La Palma Island. Volcano area. WE projected views. Sequential WE projected views of the adjusted models (2017.0-2022.0) for (incremental) deformation sources obtained from ascending and descending LOS radar data.

Title: Supplementary Movie 4.

Description: La Palma Island. Volcano area. Plan projected views. Sequential plan projected views of the adjusted models (2017.0-2022.0) for (incremental) deformation sources (only pressure and tensile sources) obtained from ascending and descending LOS radar data.

Title: Supplementary Movie 5.

Description: La Palma Island. Volcano area. SN projected views. Sequential SN projected views of the adjusted models (2017.0-2022.0) for (incremental) deformation sources (only pressure and tensile sources) obtained from ascending and descending LOS radar data.

Title: Supplementary Movie 6.

Description: La Palma Island. Volcano area. WE projected views. Sequential WE projected views of the adjusted models (2017.0-2022.0) for (incremental) deformation sources (only pressure and tensile sources) obtained from ascending and descending LOS radar data.
S
